# Molecular profiling and antimicrobial resistance of Shiga toxin-producing *Escherichia coli* O26, O45, O103, O121, O145 and O157 isolates from cattle on cow-calf operations in South Africa

**DOI:** 10.1038/s41598-019-47948-1

**Published:** 2019-08-15

**Authors:** Musafiri Karama, Alfred O. Mainga, Beniamino T. Cenci-Goga, Mogaugedi Malahlela, Saeed El-Ashram, Alan Kalake

**Affiliations:** 1 Veterinary Public Health Section, Department of Paraclinical Sciences, Faculty of Veterinary Science, University of Pretoria, Onderstepoort, South Africa; 2 Dipartimento di Scienze Biopatologiche, Laboratorio di Ispezione degli Alimenti di Origine Animale, Facoltà di Medicina Veterinaria, Università degli Studi di Perugia, Perugia, Italy; 3 School of Life Science and Engineering, Foshan University, Foshan, China; 4 Faculty of Science, Kafrelsheikh University, Kafr El-Sheikh, Egypt; 5 Gauteng Department of Agriculture and Rural Development (GDARD), Johannesburg, South Africa

**Keywords:** Microbiology, Pathogens

## Abstract

In this study, 140 cattle STEC isolates belonging to serogroups O157, O26, O145, O121, O103 and O45 were characterized for 38 virulence-associated genes, antimicrobial resistance profiles and genotyped by PFGE. The majority of isolates carried both *stx1* and *stx*2 concurrently, *stx2c*, and *stx2d*; plasmid-encoded genes *ehxA, espP, subA* and *saa* but lacked *katP* and *etpD* and *eaeA*. Possession of *eaeA* was significantly associated with the presence of *nle* genes, *katP*, *etpD*, *ureC* and *terC*. However, *saa* and *subA, stx1c* and *stx1d* were only detected in *eaeA* negative isolates. A complete OI-122 and most non-LEE effector genes were detected in only two *eaeA* positive serotypes, including STEC O157:H7 and O103:H2. The *eaeA* gene was detected in STEC serotypes that are commonly implicated in severe humans disease and outbreaks including STEC O157:H7, STEC O145:H28 and O103:H2. PFGE revealed that the isolates were highly diverse with very low rates of antimicrobial resistance. In conclusion, only a small number of cattle STEC serotypes that possessed *eaeA*, had the highest number of virulence-associated genes, indicative of their high virulence. Further characterization of STEC O157:H7, STEC O145:H28 and O103:H2 using whole genome sequencing will be needed to fully understand their virulence potential for humans.

## Introduction

Shiga toxin-producing *Escherichia coli* (STEC) are zoonotic food-borne pathogens characterized by mild to severe diarrhea, hemorrhagic colitis (HC) and the hemolytic uremic syndrome (HUS), a leading cause of acute renal failure in young children and the elderly^1^. Cattle are the major reservoir of STEC, and humans acquire STEC infections through ingestion of contaminated food of cattle origin^2^. STEC serogroups O26, O45, O103, O111, O121, O145, and O157 are frequently associated with severe illness and outbreaks in humans^3^, and colloquially termed the “top or big 7”.

The ability of STEC to cause disease in humans depends on a number of virulence factors. Bacteriophage-encoded Shiga toxins (Stx1 and Stx2) are the main STEC virulence factors^4^. Furthermore, a number of *stx1* and *stx2* Shiga toxin subtypes (15) have been described, including four *stx1* subtypes, (*stx1a*, *stx1c* and *stx1d, stx1e*) and at least 11 s*tx2* subtypes have been identified: *stx2a*, *stx2b*, *stx2c*, *stx2d*, *stx2e*, *stx2f*, *stx2g, stx2h, stx2i, stx2k, stxl*^5–7^. (http://old.iss.it/binary/vtec/cont/STEC_2018_Wrap_up.pdf). The *stx1a* and *stx2a* genes represent prototypic *Stx1* and *Stx2* toxins subtypes repectively^5^.

Another important virulence factor of STEC is intimin (*eaeA*)^8^. Intimin is encoded on the locus of enterocyte effacement (LEE), and is responsible for intimate attachment of eaeA-positive STEC strains to the host intestinal mucosa and formation of typical attaching and effacing (A/E) lesions commonly observed in STEC disease^8^. Intimin is mainly present in a subset of STEC that are involved in severe disease and have been termed enterohemorrhagic *E. coli* (EHEC).

STEC possess a number of plasmid-encoded virulence-associated genes, including enterohemolysin (*ehxA*)^9^, catalase-peroxidase (*katP*)^10^, extracellular serine protease (*esp*P)^11^ and a type II secretion system (*etpD*)^12^. Plasmid-encoded virulence-associated genes enhance pathogenicity and contribute to STEC survival in humans. Enterohemolysin is a heat labile pore-forming toxin, which lyses human erythrocytes with subsequent release of iron from heme. Possession of enterohemolysin (*ehxA*) by a STEC strain has been associated with HUS^13^. The type II secretion system facilitates protein transport across the outer membrane^14^. The extracellullar serine protease autotransporter cleaves coagulation factor V and enhances haemorrhage in HC^12^. Catalase-peroxidase defends the bacterial cell against oxidative damage by host macrophages^10^. Other plasmid-encoded virulence-associated genes include a subtilase cytotoxin (*sub*A) and the STEC autoagglutinating adhesin (Saa) (Paton *et al*., 2001). SubA suppresses the host’s immune system^15^ and facilitates STEC adherence to enterocytes. Both *subA* and *saa* are mainly observed in *eaeA* negative STEC strains^16^.

Several STEC O-islands (OIs), including OI-122, OI-57, OI-71 OI-36 and OI-43/48 encode genes, which are absent in nonpathogenic *E. coli* and are considered STEC virulence-associated genes^17^. These genes have been used in molecular risk assessment studies to classify STEC serotypes into different seropathotypes based on whether a particular serotype has been implicated in mild, severe illness or no disease at all in humans^18,19^. OI-122 carries *pagC* (PhoP-activated gene C)^20,21^, *sen* (*Shigella flexneri* enterotoxin 2) (*Z4326*)^22^, *efa1* (EHEC factor for adherence) (*Z4332)*, and *efa1* (*Z4333)*^23^. OI-122 marker genes encode proteins that play a role in immunomodulation, adhesion to host enterocytes and survival in the host^23^. Furthermore, various genes located on OI-43/48 carry virulence factors^24,25^: *iha* (IrgA homologue adhesin) encodes an adhesin^24,25^, while *ter*C and *ureC* encode tellurite resistance and urease, respectively^26^. Tellurite resistance aids bacteria in general stress response within the host environment^26^, while urease facilitates infection by lowering the STEC infective dose and enhancing bacterial survival in the host^27,28^.

STEC secrete effector proteins that are carried on a number of pathogenicity islands (PAIs) other than the LEE. These proteins have been termed “non-LEE effector proteins” (*nles* genes) because they are not encoded on the LEE pathogenicity island^29^. Important non-LEE effectors (*nles*) are located on OI-122 (*nleB*, *nleE* and *ent/espL2*), OI-57 (*nleG2-3, nleG6-2* and *nleG5-2)*, OI-71 (*nleA, nleF, nleG*, *nleH1-2, nleG2-1* and *nleG9*) and OI-36 (*nleC*, *nleD, nleB2* and *nle*H1-1)^19,30–32^. Non-LEE effectors have been associated with different functions including immunosuppression, adherence, invasion, colonization of host enterocytes, disruption of tight junctions and protein trafficking in the host^18,19,30,33^.

*Escherichia coli* strains are considered indicators of antimicrobial resistance. *E. coli* strains including STEC have been used for monitoring and surveillance of antimicrobial resistance in animals, various environments and humans. A number of studies have documented antimicrobial resistance among STEC isolates from cattle^34–37^. The emergence and spread of antimicrobial resistant *E. coli* strains has become a public health concern world-wide, as antimicrobial resistant STEC may be transferred from cattle to humans along the food chain, through occupational exposure or manure runoff from cattle farms. Monitoring of antimicrobial resistance in STEC provides information on antimicrobial abuse and the dynamics of transmission and development of antimicrobial resistant pathogens.

The first association of STEC with human disease in South Africa was reported in 1990^38^. Later on, in 1992 a large outbreak was documented in South Africa and neighboring Swaziland after affected humans had consumed water, which had been contaminated by dead cattle carcasses after a long drought^39,40^. However, information on virulence characteristics, antimicrobial resistance profiles and genotypes of cattle STEC isolates from South Africa remains scanty. The overall aim of this study was to characterize STEC serotypes of cattle origin and assess their virulence potential for humans. One hundred and forty STEC isolates belonging to serogroups O26, O45, O103, O121, O145 and O157 were screened for a number of virulence-associated genes, and antimicrobial resistance profiles. In addition, pulsed-field gel electrophoresis was used to subtype and determine relatedness/diversity among STEC isolates.

## Material and Methods

### Bacterial strains

One hundred and forty (N = 140) STEC isolates representing 33 O:H STEC serotypes, which had been previously recovered from cattle on five cow-calf operations in two provinces of South Africa were characterized in this study (Mainga *et al*., 2018). The collection included **STEC O26 serotypes:** O26:H2 (20), O26:H4 (1), O26:H7 (3), O26:H8 (8), O26:H11 (3), O26:H16 (2), O26:H19 (2), O26:H21 (7), O26:H28 (2), O26:H38 (2), O26:H45 (1) and O26:HNT (4); **STEC O45 serotypes:** O45:H2 (1), O45:H8 (3), O45:H11 (8), O45:H16 (3), O45:H19 (3), O45:H21 (2), O45:H28 (1), O45:H38 (5) and O45:HNT (12); **STEC O103 serotypes:** O103:H2 (1) and O103:H21(1); **STEC O121 serotypes:** O121:H8 (8), O121:H21 (1) and O121:HNT (1); **STEC O145 serotype**s: O145:H2 (1), O145:H7 (1), O145:H8 (1), O145:H11 (1), O145:H19 (13), O145:H28 (3) and O145:HNT (3); and **STEC O157 serotypes:** O157:H2 (1), O157:H7 (9), O157:H19 (1) and O157:H28 (1).

### DNA extraction

Frozen STEC cultures (−80 °C) were propagated aerobically overnight at 37 °C on Luria Bertani (LB) agar (Difco^TM^ Becton and Dickson & Company). Bacterial DNA was extracted using the boiling method as described previously with slight modifications^41^. Briefly, a loopful of bacterial cells was suspended into 1000 µL of sterile FA buffer (Bacto^TM^ FA Buffer, Becton and Dickson Company) in a 1.5 mL Eppendorf tube, mixed by vortexing and centrifuged at 12,000 rpm for 5 min. The supernatant was discarded and bacterial cells were re-suspended in 1000 µL of sterile FA buffer and centrifuged two more times. After the third centrifugation cycle, the supernatant was discarded. The pellet was re-suspended in 500 µL of sterile distilled water and boiled (heating block) for 20 min and cooled on ice for 10 min. Finally, the suspension was centrifuged at 12,000 rpm for 5 min, and DNA was stored at −20 °C for further use in PCR reactions.

### Detection of *stx1, stx2, eaeA* and *ehxA* genes by PCR

A multiplex polymerase chain reaction (mPCR) was carried out to detect the *stx1, stx2, eaeA* and *ehxA* genes using previously described primers (Table 1) and cycling conditions^42^. Briefly, each PCR reaction (25 µL) contained 2.5 μL of 10X Thermopol reaction buffer, 2.0 μl of 2.5 mM dNTPs, 0.25 μl of 100 mM MgCl_2_, 0.3 µM of each primer, 1U of Taq DNA Polymerase (New England BioLabs® *Inc*.) and 5 μl of DNA template. Sterile distilled water was used to top up the reaction volume to 25 µL. STEC O157:H7 EDL933 and sterile water were used as positive and negative controls, respectively. All PCR reagents were supplied by New England BioLabs (NEB, USA) except for primers, which were obtained from Inqaba Biotec (South Africa). PCR reactions were carried out in a C1000 Touch^TM^ (Bio-Rad, USA) or Veriti 96-well Thermal Cycler (Applied Biosystems, USA). PCR amplicons were electrophoresed in 2% (w/v) agarose gels in TAE (Tris–acetate-ethylenediamine tetraacetic acid) buffer, stained with ethidium bromide (0.05 mg/μl) and visualized under ultraviolet (UV) light with a Gel Doc system (Bio-Rad, USA).

**Table 1:** DNA oligonucleotides used in the analysis of STEC by PCR.

Gene Location	Target Gene	Sequence (5′ to 3′)	Amplicon Size (bp)	References
Bacteriophage-encoded genes	*stx1*	**F:** ATAAATCGCCATTCGTTGACTAC **R:** AGAACGCCCACTGAGATCATC	180	(Paton and Paton^42^)
	*stx2*	**F:** GGCACTGTCTGAAACTGCTCC **R:** TCGCCAGTTATCTGACATTCTG	255	(Paton and Paton^42^)
	*stx1c*	**F1:** CCTTTCCTGGTACAACTGCGGTT **R1:** CAAGTGTTGTACGAAATCCCCTCTGA	252	(Scheutz *et al*.^5^)
	*stx1d*	**F1:** CAGTTAATGCGATTGCTAAGGAGTTTACC **R2:** CTCTTCCTCTGGTTCTAACCCCATGATA	203	(Scheutz *et al*.^5^)
	*stx2a*	**F2:** GCGATACTG**R**G**B**ACTGTGGCC **R3:** CCG**K**CAACCTTCACTGTAAATGTG	349	(Scheutz *et al*.^5^)
	*stx2c*	**F1:** GAAAGTCACAGTTTTTATATACAACGGGTA **R2:** CCGGCCACYTTTACTGTGAATGTA	177	(Scheutz *et al*.^5^)
	*stx2d*	**F1:** AAARTCACAGTCTTTATATACAACGGGTG **R1:** TTYCCGGCCACTTTTACTGTG **O55-R:** TCAACCGAGCACTTTGCAGTAG	179 235	(Scheutz *et al*.^5^)
	*stx2e*	**F1:** CGGAGTATCGGGGAGAGGC **R2:** CTTCCTGACACCTTCACAGTAAAGGT	411	(Scheutz *et al*.^5^)
	*stx2f*	**F1:** TGGGCGTCATTCACTGGTTG **R1:** TAATGGCCGCCCTGTCTCC	424	(Scheutz *et al*.^5^)
	*stx2g*	**F1:** CACCGGGTAGTTATATTTCTGTGGATATC **R1:** GATGGCAATTCAGAATAACCGCT	573	(Scheutz *et al*.^5^)
	*eaeA*	**F:** GACCCGGCACAAGCATAAGC **R:** CCACCTGCAGCAACAAGAGG	384	(Paton and Paton^42^)
Plasmid-encoded genes	*ehxA*	**F:** GCATCATCAAGCGTACGTTCC **R:** AATGAGCCAAGCTGGTTAAGCT	584	(Paton and Paton^42^)
	*katP*	**wkat-F:** AACTTATTTCTCGCATCATCC **wkat-B:** CTTCCTGTTCTGATTCTTCTGG	2125	(Brunder *et al*.^10^)
	*espP*	**F:** AAACAGCAGGCACTTGAACG **R:** GGAGTCGTCAGTCAGTAGAT	1830	(Brunder *et al*.^12^)
	*etpD*	**D1-** CGTCAGGAGGATGTTCAG **D13R-** CGACTGCACCTGTTCCTGATTA	1062	(Schmidt *et al*.^14^)
	*saa*	**F:** CGTGATGAACAGGCTATTGC **R:** ATGGACATGCCTGTGGCAAC	119	(Paton and Paton, 2002)
	*subA*	**SubHCDF**: TATGGCTTCCCTCATTGC C **SubSCDR:** TATAGCTGTTGCTTCTGACG	556	(Paton and Paton, 2005)
Pathogenicity Island-encoded genes				
OI-71	*nleA (Z6024)*	**F:** ATGAACATTCAACCGACCATAC **R:** GACTCTTGTTTCTTGGATTATATCAAA	1296	(Coombes *et al*.^19^)
OI-122	*nleB (Z4328)*	**F:** GGAAGTTTGTTTACAGAGACG **R:** AAAATGCCGCTTGATACC	297	(Coombes *et al*.^19^)
OI-36	*nleB2 (Z0985)*	**F:** GTTAATACTAAGCAGCATCC **R:** CCATATCAAGATAGATACACC	475	(Coombes *et al*.^19^)
OI-36	*nleC (Z0986)*	**F:** ACAGTCCAACTTCAACTTTTCC **R:** ATCGTACCCAGCCTTTCG	777	(Coombes *et al*.^19^)
OI-36	*nleD (Z0990)*	**F:** GGTATTACATCAGTCATCAAGG **R:** TTGTGGAAAACATGGAGC 426	426	(Coombes *et al*.^19^)
OI-122	*nleE (Z4329)*	**F:** GTATAACCAGAGGAGTAGC **R:** GATCTTACAACAAATGTCC	260	(Coombes *et al*.^19^)
OI-71	*nleF (Z6020)*	**F:** ATGTTACCAACAAGTGGTTCTTC **R:** ATCCACATTGTAAAGATCCTTTGTT	567	(Coombes *et al*.^19^)
OI-71	*nleG (Z6010)*	**F:** ATGTTATCGCCCTCTTCTATAAAT **R:** ACTTAATACTACACTAATAAGATCCA	902	(Coombes *et al*.^19^)
OI-71	*nleG2-1 (Z6025)*	**F:** ACCAGAAACCTGACTTCG **R:** CAGCATCTTCATATACTACAGC	406	(Coombes *et al*.^19^)
OI-57	*nleG2-3*	**F:** GGATGGAACCATACCTGG **R:** CGCAATCAATTGCTAATGC	551	(Coombes *et al*.^19^)
OI-57	*nleG5-2*	**F:** TGGAGGCTTTACGTCATGTCG **R:** CCGGAACAAAGGGTTCACG	504	(Coombes *et al*.^19^)
OI-57	*nleG6-2*	**F:** CGGGTCAGTGGATGATATGAGC **R:** AAGTAGCATCTAGCGGTCGAGG	424	(Coombes *et al*.^19^)
OI-71	*nleG9 (Z2560)*	**F:** GTTCGTGCCCGAATTGTAGC **R:** CACCAACCAAACGAGAAAATG	409	(Coombes *et al*.^19^)
OI-71	*nleH1-2 (Z6021)*	**F:** AACGCCTTATATTTTACC **R:** AGCACAATTATCTCTTCC	589	(Coombes *et al*.^19^)
OI-36	*nleH1-1 (Z0989)*	**F:** GTTACCACCTTAAGTATCC **R:** GTTTCTCATGAACACTCC	456	(Coombes *et al*.^19^)
OI-122	*ent/espL2*	**F:** GAATAACAATCACTCCTCACC **R:** TTACAGTGCCCGATTACG	433	(Coombes *et al*.^19^)
OI-122	*Efa1 (Z4332)*	**Z4321-a:** ATGAGTGGTTCAAGACTGG **Z4321-b:** CCAACTCCAACAGTAAATCC	521	(Karmali *et al*.^18^)
OI-122	*Efa1 (Z4332)*	**Z4326-a:** GGATGGAACCATACCTGG **Z4326-b:** CGCAATCAATTGCTAATGC	551	(Karmali *et al*.^18^)
OI-122	*sen (Z4326)*	**Z4332-a:** CTCCCAGAGATAATTTTGAGG **Z4332-b:** CAACTGTATGCGAATAGTACTC	504	(Karmali *et al*.^18^)
OI-122	*pagC*	**Z4333-a:** CTGTCAGACGATGACATTGG **Z4333-b:** GAAGGATGGGCATTGTGTC	547	(Karmali *et al*.^18^)
OI-43/48	*ure*C	**F:** TCT AAC GCC ACA ACC TGT AC **R:** GAG GAA GGC AGA ATA TTG GG	397	(Nakano *et al*.^27^)
OI-43/48	*Ter-island*	**F:** GAC AAA CTC TCC GGG ATA ACT CA **R:** TGC GGG TGC TGG TGT GGG ATA A	356	(Taylor *et al*.^26^)
OI-43/48	*iha*	**Iha-I:** CAG TTC AGT TTC GCA TTC ACC **Iha-II:** GTA TGG CTC TGA TGC GAT G	1305	(Janka *et al*.^44^)

### Detection of genes encoding Shiga toxin (*stx*) subtypes

To detect *stx1c, stx1d*, *stx2a*, stx*2c*, stx*2d* genes, single PCR assays were performed using primers (Table 1) and cycling conditions described elsewhere (Scheutz *et al*., 2012) (Table 1). Briefly, each PCR reaction (25 µL) contained 2.5 μL of 10X Thermopol reaction buffer, 2.0 μl of 2.5 mM dNTPs, 0.25 μl of 100 mM MgCl_2_, 0.3 µM final of each primer concentration, 1U of Taq DNA Polymerase (New England BioLabs® Inc.) and 5 μl of DNA template.

### Detection of plasmid and pathogenicity island encoded genes

Primers (Table 1) and cycling conditions described in previous studies were used to amplify virulence-associated genes located on plasmids and pathogenicity islands. Amplification reactions for *ehxA*, *saa*, *sub*A^16,42,43^*, kat*P^11^, *esp*P^12^, and *etpD*^14^ genes were conducted in singleplex PCR reactions. Amplification of OI-122 gene markers including *pag*C (*Z4321*), *sen* (*Z4326*), *efa1* (*Z4332* and *Z4333*) was carried out as previously described (Karmali *et al*., 2003). Amplification of non-LEE-encoded effector (*nle*) genes including *nleA*, *nleB*, *nleB*_*2*_, *nleC*, *nleD*, *nleE*, *nleF*, *nleG*, *nleG2-1*, *nleG2-3*, *nleG5-2*, *nleG6-2*, *nleG9*, *nleH1*, *nleH2*, and *ent/espL2* were performed in singleplex PCR reactions according to Coombes *et al*.^19^. PCR reactions for OI-43/48 island markers, *iha*, *ter-*island and *ure*C, were also carried out according to previous studies^26,27,44^. STEC O157:H7 EDL933 and sterile distilled water were used as positive and negative controls, respectively.

### Antimicrobial susceptibility testing

All the 140 STEC isolates were tested for resistance against a panel of 15 antimicrobials by the disk diffusion method as described by the Clinical and Laboratory Standards Institute (CLSI, 2014). Briefly, pure STEC colonies were inoculated on Mueller Hinton agar (MHA) (Oxoid, UK) and incubated overnight at 37 °C. Bacterial suspensions (0.5 McFarland) of overnight cultures were prepared in 0.85% physiological saline. A sterile cotton swab was used to inoculate MHA plates to achieve a confluent growth. Antimicrobial discs were placed on inoculated MHA plates by means of a BBL Sensi-disk or Oxoid disk dispenser and incubated aerobically at 37 °C ± 2 °C for 18 h. The panel of 15 antimicrobials consisted of amoxicillin-clavulanic acid (20 µg–10 µg), amikacin (30 µg), ampicillin (10 µg), ceftazidime (30 µg), cephalothin (30 µg), cefoperazone (75 µg), ceftriaxone (30 µg), chloramphenicol (30 µg), ciprofloxacin (5 µg), gentamicin (10 µg), kanamycin (30 µg), nalidixic acid (30 µg), trimethoprim-sulfamethoxazole (1.25 µg and 23.75 µg, respectively) and tetracycline (30 µg). Antimicrobial disks were obtained from Becton Dickinson (BD, USA) and Oxoid (Thermo Scientific, UK), respectively. *Escherichia coli* ATCC 25922 was used as the control strain. Isolates were classified as susceptible, intermediate or resistant to each antimicrobial agent according to the CLSI interpretative criteria. However, in the final analysis, intermediate readings were assigned to the resistant category.

### Pulsed-field gel electrophoresis

To subtype STEC isolates, DNA was extracted, digested with the *Xba*I restriction enzyme and subjected to PFGE according to the CDC/PulseNet protocol (https://www.cdc.gov/pulsenet/pdf/ecoli-shigella-salmonella-pfge-protocol-508c.pdf). *Salmonella* enterica serotype Braenderup (strain H9812; American Type Culture Collection, BAA-664) DNA was used as the molecular weight marker. PFGE fingerprints were analyzed for similarity, and a dendrogram was generated by Bionumerics software, version 6.6 (Applied Maths, Sint Martens-Latem, Belgium) with the Dice similarity indices (complete linkage; optimization, 1.5%; position tolerance, 1.5%) and the unweighted-pair group method with arithmetic means (UPGMA).

### Statistical analysis

Descriptive statistical analysis was performed using the statistical package for social sciences (SPSS) software version 21 (IBM® SPSS® Statistics 21). Fisher’s exact test was used to determine if there were statistically significant differences and associations between gene proportions. *P* values of < 0.05 were considered statistically significant.

## Results

### Virulence-associated genes

The distribution of *stx*-encoding virulence genes (N = 140) was as follows: 4.3% of isolates carried *stx1* only, 34.3% carried *stx2* only, and 61.4% carried both *stx1* and *stx2.* Among the 92 *stx1* positive isolates, 20.7% carried *stx1c* and 18.5% were *stx1d* positive; 6.5% possessed both *stx1c* + *stx1d.* The *stx1c* and *stx1d* subtypes were significantly (*P* < 0.05) detected in STEC O26 and STEC O45 serogroups. Of the 134 *stx2* positive isolates, *stx2* subtypes were distributed as follows: 91.8%, 97%, and 56% carried *stx2a, stx2c*, and *stx2d*, respectively (Table 2). The most common toxin gene combinations among *stx2*-positive isolates were *stx2a* + *stx2c* + *stx2d*, 37.1%, *stx2c* + *stx2d*, 35%, *stx2c* + *stx2d*, 5.7%, and *stx1c* + *stx2a* + *stx2c* + *stx2d* in 5% of isolates (Table 2). All isolates were negative for *stx2e*, *stx2f* and *stx2g* subtypes.

**Table 2:** Distribution of *stx* subtypes among STEC isolates.

SEROTYPEs	No. of tested Isolates	*stx1c* n = 92	*stx1d* n = 92	*stx2a* n = 134	*stx2c* n = 134	*stx2d* n = 134	*stx* subtype combinations
**O26:H2**	1	+	−	+	+	−	*stx1c, stx2a, stx2c*
**O26:H2**	4	+	−	+	+	+	*stx1c, stx2a, stx2c, stx2d*
**O26:H2**	2	−	+	−	−	−	*stx1d*
**O26:H2**	2	−	+	+	+	+	*stx1d, stx2a, stx2c, stx2d*
**O26:H2**	2	−	−	+	+	−	*stx2a, stx2c*
**O26:H2**	9	−	−	+	+	+	*stx2a, stx2c, stx2d*
O26:H4	1	−	+	+	−	−	*stx1d, stx2a*
**O26:H7**	1	−	+	−	−	−	*stx1d*
**O26:H7**	1	−	−	+	+	+	*stx2a, stx2c, stx2d*
**O26:H7**	1	−	−	+	+	+	*stx2a, stx2c, stx2d*
**O26:H8**	1	−	+	+	+	−	*stx1d, stx2a, stx2c*
**O26:H8**	3	−	−	+	+	−	*stx2a, stx2c*
**O26:H8**	4	−	−	+	+	+	*stx2a, stx2c, stx2d*
**O26:H11**	2	−	−	+	+	−	*stx2a, stx2c*
**O26:H11**	1	−	−	+	+	+	*stx2c, stx2d*
O26:H16	1	−	−	+	+	−	*stx2a, stx2c*
O26:H16	1	−	−	+	+	+	*stx2a, stx2c, stx2d*
O26:H19	1	−	−	+	+	+	*stx2a, stx2c, stx2d*
O26:H19	1	−	−	+	+	+	*Stx2a, stx2c, stx2d*
O26:H21	3	−	−	+	+	−	*stx2a, stx2c*
**O26:H21**	4	−	−	+	+	+	*stx2a, stx2c, stx2d*
O26:H28	1	+	−	+	+	−	*stx1c, stx2a, stx2c*
O26:H28	1	−	+	−	−	−	*stx1d*
O26:H38	1	−	−	+	+	−	*stx2a, stx2c*
O26:H38	1	−	−	+	+	+	*stx2a, stx2c, stx2d*
O26:H45	1	−	−	+	+	−	*stx2a, stx2c*
O26:HNT	1	−	−	+	+	−	*stx2a, stx2c*
O26:HNT	3	−	−	+	+	+	*stx2a, stx2c, stx2d*
**O45:H2**	1	+	+	+	+	+	*stx1c, stx1d, stx2a, stx2c, stx2d*
O45:H8	1	+	−	−	−	−	*stx1c*
O45:H8	1	+	+	−	−	−	*stx1c, stx1d*
O45:H8	1	−	−	+	+	−	*stx2a, stx2c*
O45:H11	3	+	+	+	+	+	*stx1c, stx1d, stx2a, stx2c, stx2d*
O45:H11	2	−	+	+	+	+	*stx1d, stx2a, stx2c, stx2d*
O45:H11	3	−	−	+	+	+	*stx2a, stx2c, stx2d*
O45:H16	2	−	−	+	+	−	*stx2a, stx2c*
O45:H16	1	−	−	+	+	+	*stx2a, stx2c, stx2d*
O45:H19	1	+	−	+	+	+	*stx1c, stx2a, stx2c, stx2d*
O45:H19	2	−	−	+	+	+	*stx2a, stx2c, stx2d*
O45:H21	2	−	−	+	+	−	*stx2a, stx2c*
O45:H28	1	+	+	−	−	−	*stx1c, stx1d*
O45:H38	5	−	−	+	+	+	*stx2a, stx2c, stx2d*
O45:HNT	2	+	−	+	+	−	stx1c*, stx2a, stx2c*
O45:HNT	2	+	−	+	+	+	*stx1c, stx2a, stx2c, stx2d*
O45:HNT	5	−	−	+	+	−	*stx2a, stx2c*
O45:HNT	3	−	−	+	+	+	*stx2a, stx2c, stx2d*
**O103:H2**	1	−	+	−	−	−	*stx1d*
**O103:H21**	1	−	−	+	+	−	*stx2a, stx2c*
**O121:H8**	4	−	−	+	+	−	*stx2a, stx2c*
**O121:H8**	2	−	−	+	+	+	*stx2a, stx2c, stx2d*
**O121:H8**	1	−	−	+	+	−	*stx2c*
**O121:H8**	1	−	−	+	+	+	*stx2a, stx2c, stx2d*
O121:H21	1	−	−	+	+	+	*stx2a, stx2c, stx2d*
O121:HNT	1	−	−	+	+	+	*stx2a, stx2c, stx2d*
O145:H2	1	−	−	+	+	−	*stx2a, stx2c*
**O145:H7**	1	−	−	+	+	+	*stx2a, stx2c, stx2d*
O145:H8	1	−	−	+	+	−	*stx2a, stx2c*
O145:H11	1	−	−	+	+	+	*stx2a, stx2c, stx2d*
O145:H19	1	+	−	+	+	−	*stx1c, stx2a, stx2c*
O145:H19	12	−	−	+	+	−	*stx2a, stx2c*
**O145:H28**	2	−	−	+	+	−	*stx2a, stx2c*
**O145:H28**	1	−	−	+	+	+	*stx2a, stx2c, stx2d*
O145:HNT	2	−	−	+	+	−	*stx2a, stx2c*
O145:HNT	1	−	−	+	+	+	*stx2a, stx2c, stx2d*
O157:H2	1	−	−	+	+	+	*stx2a, stx2c, stx2d*
**O157:H7**	7	−	−	+	+	+	*stx2a, stx2c, stx2d*
**O157:H7**	2	−	−	+	+	+	*stx2c, stx2d*
O157:H19	1	−	−	+	+	−	*stx2a, stx2c*
O157:H28	1	−	−	+	+	−	*stx2a, stx2c*
**TOTAL**	**140**	**19**	**17**	**123**	**131**	**75**	
**% Positive**		**20,7**	**18,5**	**91,8**	**97,8**	**56**	

^a^Serotypes in **bold** have been identified previously as human pathogens causing diarrhea, bloody diarrhea and HUS.

The *eaeA* gene was detected in only 12.1% of isolates. Among the 17 STEC isolates, which carried *eaeA*, nine possessed also *stx2a* + *stx2c* + *stx2d* and five had *stx2a* + *stx2c* concurrently (Table S1). However, all isolates that were *eaeA* positive, lacked *saa*, *stx1c* and *stx1d*. The *eaeA* gene was present in STEC O157:H7 (9 isolates), STEC O157:H28 (1 isolate), STEC O26:H2 (2 isolates), STEC O103:H2 (1 isolate), STEC O145:H28 (3 isolates) and STEC O145:HNT (1 isolate) (Table S1) isolates only.

**Figure 1:**
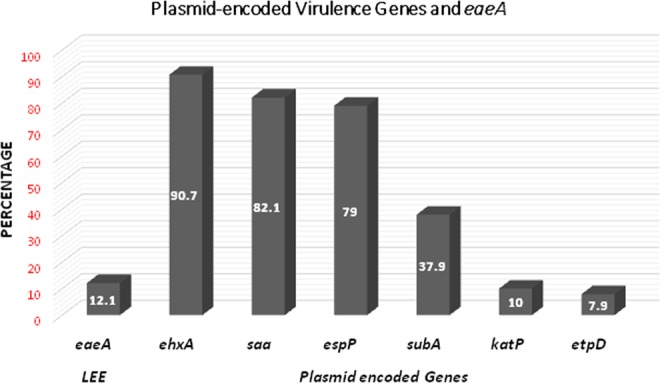
Relationship between plasmid-encoded Virulence Genes and *eaeA*.

The following rates were observed for plasmid-encoded genes (Table S2, Fig. 1): *ehxA*, 90.7%; *subA*, 85%; *saa*, 82.1%; *espP*, 79.3%; *katP*, 10% and *etpD*, 7.9% (Fig. 1). All the 14 *katP* positive isolates were also *eaeA* positive. The *katP* and *etpD* genes were significantly (*P* < 0.05) observed in *eaeA* positive isolates (*P* < 0.000). In addition to *eaeA*, all O157:H7 isolates possessed the full complement of plasmid markers, including *ehxA*, *sub*A*, katP, espP* and *etpD* except *saa*. However, STEC O145:H28, O145: HNT and O157:H28 that were also *eaeA* positive, carried *ehxA*, *sub*A, *katP* and *espP* but lacked *etpD*. The *katP*/*eaeA* genotype was observed in 13/17 isolates, including STEC O145:H28 (3), STEC O145:HNT (1), STEC O157:H7 (9) and STEC O157:H28 (1) whereas the *etpD/eaeA* genotype was present in 10/17 isolates, including STEC O103:H2 (1), and STEC O157:H7 (9) isolates (Table S1).

**Figure 2:**
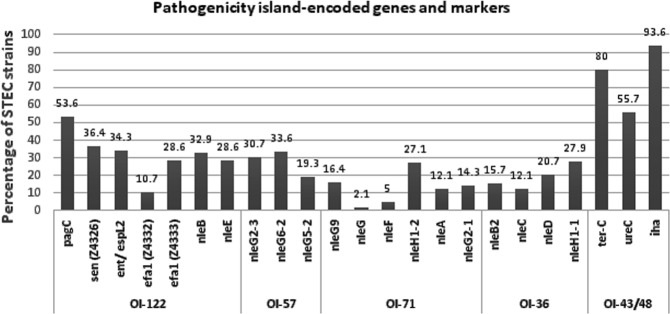
Distribution of pathogenicity island-encoded genes.

The distribution of OI-122 markers was as follows: *pagC*, 53.6%; *sen*, 34.3%; *Z4332*, 10.7%; and *Z4333*, 28.6% (Table S2, Fig. 2). All OI-122 (full complement OI-22 genes) markers were observed in only 7.1% of isolates, which belonged two only serotypes, including O157:H7 (9 isolates) and O103:H2 (1 isolate). An incomplete OI-122 was observed in 60% of isolates and 32.9% carried none of OI-122 markers. OI-43/48 encoded genes were distributed as follows: *iha*, 93.6%; *terC*, 80%; and *ureC*, 55.7% (Table S2, Fig. 2). All OI-43/48 markers were detected in 52.9% of isolates. Both *terC* (P = 0.032) and *ureC* (*P* < 0.000) were significantly (*P* < 0.05) prevalent among *eaeA*-positive STEC isolates. However, 2.1% (3/140) of isolates were negative for all OI-43/48 markers (Table S2).

The following proportions were observed for non-LEE effector (*nle*) genes: ent/espL2, 34.3%; *nleB*, 32.9%; *nleE*, 28.6%; *nleG2-3*, 30.7%; *nleG6-2*, 33.6%; *nleG5-*2, 19.3%; *nleH1-*2, 27.1%; *nleG9*, 16.4%: *nleG2-1*,14.3%; *nleA*, 12.1%; *nleF*, 5.0%; *nleG*, 2.1%; *nleH1-1*, 27.9% (39/140); *nleD*, 20.7%; *nleB2*,15.7%; and *nleC*, 12.1% (Table S2 and Fig. 2). More than 10 *nle* genes were observed in 12.9% of the isolates, which were mainly *eaeA* positive, eight to nine *nle* genes were present in 6.4%, and one to seven *nle* genes were detected in 30.7% of the isolates. The remaining 50% of isolates did not carry a non-LEE effector gene.

Overall, the highest number of virulence genes (more than 30 genes) was detected in STEC O157:H7 isolate. STEC O145:H28/HN isolate had 25–30 genes, a number of STEC O45 isolates (H21, H11, H2, H16, HNT) and 2 STEC O26:H2/H21 carried 20–30 virulence-associated genes.

### Antimicrobial resistance

Of the 140 STEC isolates, 97.9% were susceptible to all the 15 antimicrobials. Only 2.1% of STEC isolates were antimicrobial resistant, including one STEC O26:H11 isolate which was resistant to tetracycline, one STEC O26:H4 which was resistant to ampicillin and tetracycline and one STEC O45:H21 isolate which was resistant ampicillin, tetracycline and cephalothin.

### Pulsed field gel electrophoresis

**Figure 3:**
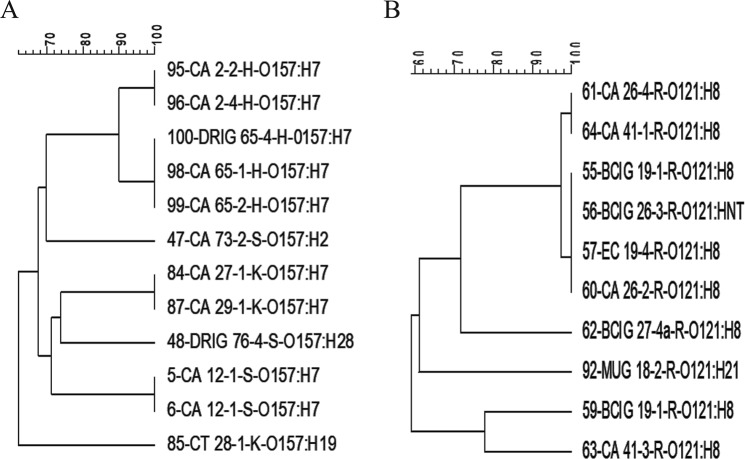
PFGE dendograms of serogroups O157 (**A**) and O121 (**B**) STEC isolates.

**Figure 4:**
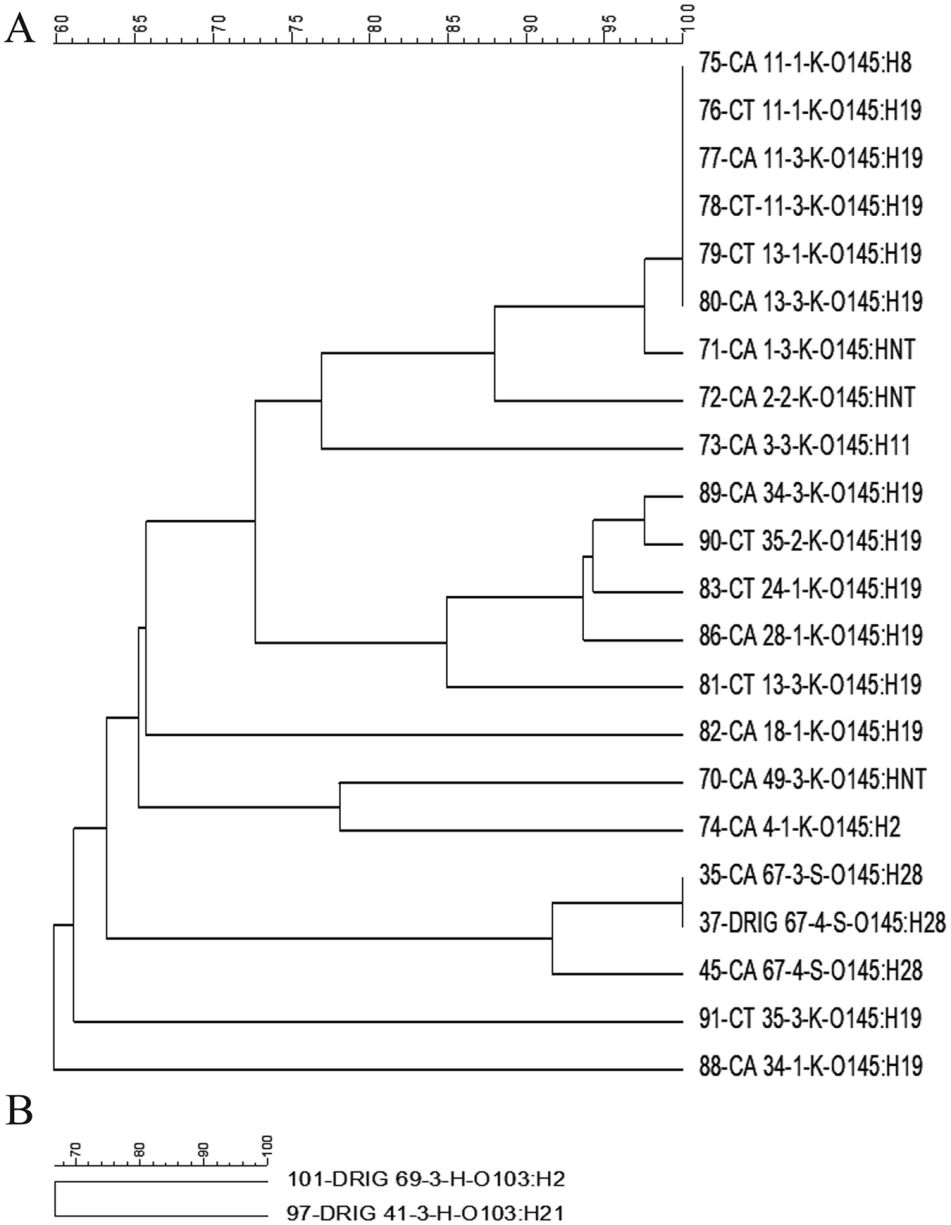
PFGE dendograms of serogroups O145 (**A**) and O103 (**B**) STEC isolates.

**Figure 5:**
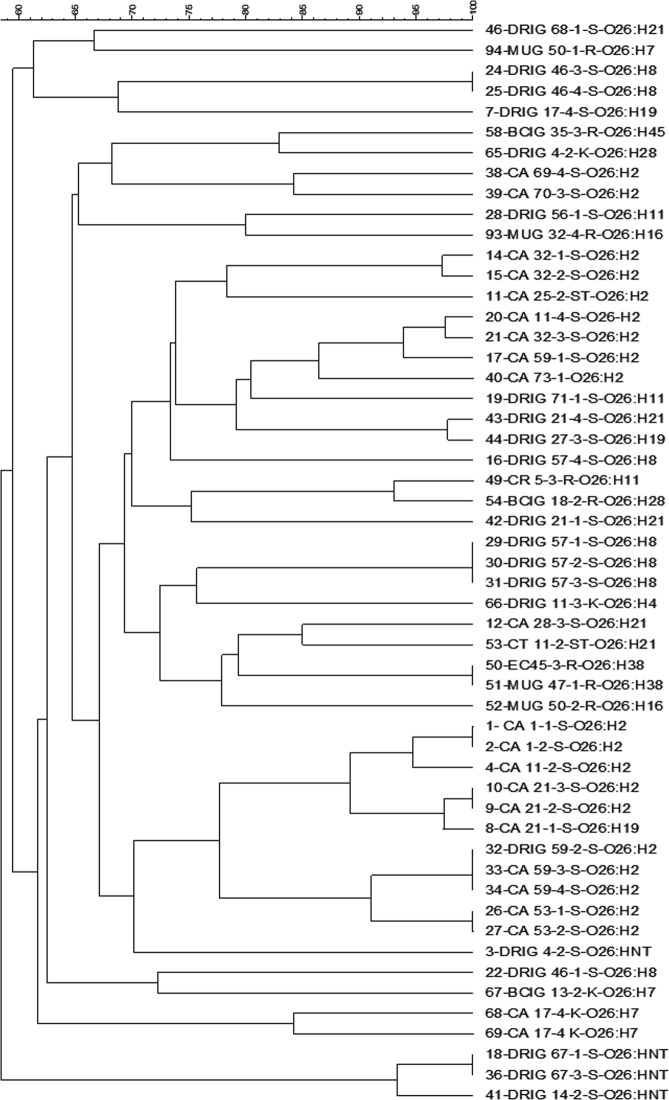
A PFGE dendogram of serogroup O26 STEC isolates.

**Figure 6:**
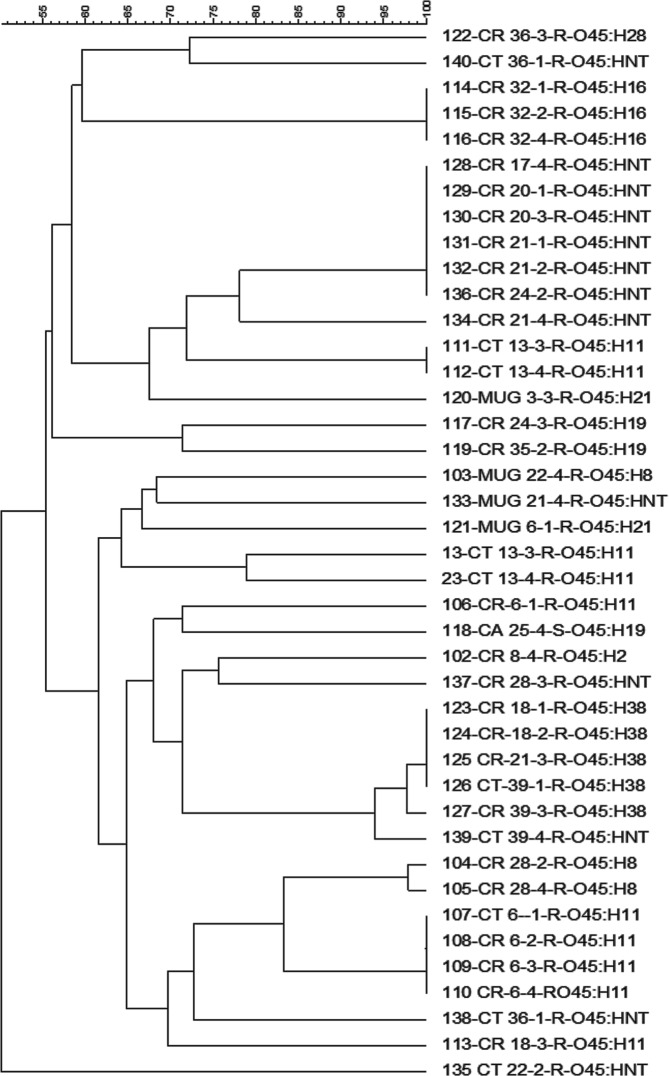
A PFGE dendogram of serogroup O45 STEC isolates.

PFGE was conducted to investigate genetic relatedness among the STEC isolates. Six dendograms (Figs 3–6) that displayed relationships among individual serogroups were generated. All the 140 isolates yielded 101 distinct pulsotypes, including 43 for STEC O26, 27 for STEC O45, 2 for STEC O103, 6 for STEC O121, 7 for STEC O157, and 16 for O145 suggesting a high diversity (Dice similarity index < 70%) among STEC isolates in different serogroups. Most of the pulsotypes represented single isolates. The 39 isolates which shared identical PFGE profiles (100% similarity) in different serogroups either belonged to the same serotype or were recovered from the same animal or farm.

## Discussion

STEC are frequently implicated in mild to severe human disease and outbreaks (EFSA, 2013). Since the first report on foodborne STEC in humans nearly 40 years ago, a number of studies have been published on virulence, antimicrobial resistance and molecular epidemiology of STEC around the world. However, most reports on STEC are based on data from high-income industrialised nations, while current studies on virulence, antimicrobial resistance and molecular epidemiology of STEC isolates from African countries including South Africa remain scanty. Cattle are a major reservoir of STEC. Molecular risk assessment studies on STEC isolates from cattle have contributed to a better understanding of the virulence potential cattle STEC present to humans and made it possible to differentiate low virulence from highly virulent STEC isolates. In this study, 140 STEC isolates from apparently healthy cattle on five cow-calf operations in South Africa were characterized for a panel 38 virulence-associated genes, antimicrobial resistance and PFGE profiles. The collection of isolates under study was a subset of a larger collection of STEC strains belonging 33 serotypes associated with serogroups O157, O45, O103, O121, O26 and O145.

The majority of STEC isolates carried both *stx1* and *stx2* concurrently. Almost all *stx2*-positive isolates (95.7%) harbored *stx2a, stx2c* and *stx2d*. Only a small fraction of isolates carried *stx1* only. The *stx2d* subtype identified in this study was the *stx2d-*activatable variant^45^. The widespread distribution of *stx2a, stx2c and stx2d* subtypes in cattle isolates is in agreement with previous studies which have reported high rates of *stx2a, stx2c* and *stx2d* subtypes among STEC isolates from cattle in comparison to different *stx1* subtypes^46–50^. STEC isolates that carry *stx2* are more virulent compared to strains that possess *stx1* alone or both *stx1* and *stx2* concurrently, and are frequently incriminated in outbreaks and severe human disease manifestations such as HC and HUS^45,51,52^. Furthermore, some studies have shown that Stx2, Stx2c and Stx2d subtypes are more potent than Stx1^45,53^. In addition, Rasooly and Do^54^ reported that Stx2 was heat stable and not inactivated at currently approved pasteurization temperatures, making Stx2-producing isolates more likely to be implicated in human STEC disease outbreaks involving pasteurized cattle dairy products.

Less than 50% of isolates carried *stx1c* and *stx1d* subtypes. The *stx1c* subtype was significantly more frequent than *stx1d*, in agreement with a number of studies which have reported that STEC isolates of cattle origin are mainly *stx1c* positive^47,49^. STEC isolates that possess *stx1c* and/or *stx1d* subtypes have been mostly implicated in asymptomatic or mild diarrhea in humans^52,55,56^. However, some reports have implicated *stx1c* positive isolates in cases of human disease showing bloody diarrhea^56–58^. Interestingly, STEC O45:H2 and STEC O45:H11 isolates possessed *stx1c, stx1d, stx2, stx2c*, and *stx2d* concurrently. While STEC O45:H2 is a recognized enterohemorrhagic *E. coli*^59^, so far, there are no reports that have associated STEC O45:H11 isolates with severe human disease. Although STEC O45:H11 is not a recognised enterohemorrhagic *E. coli*, it is possible that the presence of numerous *stx* variants in this STEC serotype may be indicative of high virulence, assuming that all toxin encoding genes are maximally expressed during STEC infection in humans.

The majority of STEC isolates lacked *eaeA*, consistent with previous reports on cattle STEC, which have shown that only a subset of cattle STEC are *eaeA* positive^46,48,50^. The *eaeA* gene was present in seropathotypes A and B STEC strains (STEC O157:H7, STEC O103:H2, STEC O26:H2, STEC O145:H28) that were also *stx2c* and/or *stx2d* positive but lacked *stx1c* and *stx1d* genes. Possession of *stx2 and eaeA* genes by a STEC strain is indicative of highly virulent STEC strains (EFSA, 2013). STEC seropathotypes A and B strains are highly pathogenic for humans, and commonly implicated in outbreaks and severe disease, including HC or HUS^18,60^ worldwide.

Plasmid-encoded virulence markers (*ehxA*, *espP*, and *saa*) were present in the majority of isolates. However, *subA* (37.9%), *katP* (10%) and *etpD* (7.9%) virulence markers were less frequent (<50%). Similar reports have documented high rates of *ehxA, espP* and *saa* and very low rates of *katP* and *etpD* in STEC isolates from cattle^35,46,47,49,50,61^. However, the rates of these genes in cattle STEC isolates are variable from country to country^46,50,61–63^. Furthermore, *katP* and *etpD* were exclusively detected in *eaeA*-positive STEC O157:H7, O103:H2 and O145:H28 that were also *ehx*A*, esp*P positive. Possession of all the four plasmid-encoded genes (*katP* and *etpD, ehxA, espP*) concurrently is usually indicative of a complete plasmid (pO157 or its homologs). Carriage of a complete plasmid and *eaeA* is characteristic of highly virulent STEC isolates that are commonly incriminated in severe disease (HC and HUS) and outbreaks in humans^64–66^.

Plasmid-encoded genes *saa* and *subA* were exclusively detected among *eaeA*-negative STEC isolates only, consistent with other studies, which have reported the presence of *saa* and *subA* in *eaeA*-negative STEC isolates^46–50^. Both *saa* and *sub*A genes are widespread among STEC serotypes that have been associated with uncomplicated diarrhea (O26:H2, O26:H8, O26:H21, O121:H8, O45:H2 and O145:H8), hemorrhagic colitis (O26:H7 and O145:H7) and hemolytic uremic syndrome (O26:H11) in humans^43,67–70^.

Karmali *et al*.^18^ suggested that possession of OI-122 marker genes *pag*C*, sen*, *efa1* (*Z4332*), *efa1* (*Z4333*) is indicative of a complete OI-122. A complete OI-122 was observed in only 7.1%. that were mostly *stx2*/*eaeA*-positive. Isolates which possessed the full complement of OI-122 marker genes belonged to serotypes which are commonly associated with STEC disease outbreaks, including HUS in humans (STEC O157:H7 and STEC O103:H2). Reports that have documented a complete OI-122 among clinically relevant isolates, including STEC O157:H7 and STEC O103:H2 have suggested that the presence of a complete OI-122 and *stx2* in *eaeA*-positive strains is indicative of highly virulent STEC strains^18,68,71,72^.

Most STEC isolates that had an incomplete OI-122 were seropathotypes B and C STEC strains that are usually incriminated in mild or uncomplicated diarrhea (STEC O26:H2, O26:H8, O26:H21, O103:H21, O45:H2)^18^. However, isolates that were negative for all OI-122 markers were mainly seropathotypes D or E strains that are very rare in human disease or have never been incriminated in human disease^18^.

OI-43/48-encoded genes, including *iha*, *terC* and *ureC* are considered suitable markers of virulence in STEC serotypes which are implicated in severe human diseases and outbreaks^27,73^. OI-43/48 marker genes (*iha*, *terC* and *ureC*) were present in more than 80% of isolates in agreement with previous reports which have found that OI-43/48 marker genes (*iha, terC and ureC*) are widespread in cattle STEC^46,74–76^. However, the *ureC* gene was significantly detected in *eaeA*-positive STEC isolates (17/17) in comparison to *eaeA*-negative STEC (61/123)^28,73,75,76^. Furthermore, *ureC* positive STEC belonged to serotypes that have been incriminated in mild and severe STEC illness in humans, including STEC O26:H2, O26:H7, O26:H8, O26:H21, O45:H2, O103:H2, O145:H7, O145:H28 and O157:H7. The presence of urease genes in STEC has been associated with adherence and survival of bacteria within acidic environments in the host^25,77^ particularly in STEC serotypes that have been implicated in severe human disease^27,73^. The *iha* gene product is considered an additional adhesin in STEC strains^24^. Although the role of tellurite resistance genes in STEC virulence remains unclear, it has been hypothesized that tellurite resistance genes may promote adherence, STEC survival in the host, and resistance against pore-forming colicins and bacteriophage (T5) infection^25^.

A number of clinically relevant *eaeA*-positive STEC strains, including STEC O157:H7, STEC O145:H28, STEC O103:H2, STEC O26:H2 possessed the majority of *nle* genes. These isolates carried also the *nle* ‘virulence gene signature’, which includes of *nleB*, *nleE*, *ent/espL2*, *nleG2-3, nleG5-2*, *nleG9*, *nleG2-1* and *nleB2* concurrently^18,19,64,65^. However, clinically relevant *eaeA*-negative STEC serotypes, including O26:H2, O26:H21, O157:H19, O45:H11, O45:H16 and O45: HNT STEC isolates possessed 9 to 11/15 *nle*-encoding genes. While O26:H2 and O26:H21 strains have been previously implicated in mild diarrhea in humans^59,60^, STEC O157:H28 and O45:H11 have never been incriminated in human disease. The high proportion of *nle-*encoding genes in STEC O157:H19, O45:H11, O45:H16 and O45:HNT that have never been implicated in human disease may be an indication of emerging virulent cattle STEC strains that should be closely monitored in this part of the world as they may be high risk STEC serotypes with potential to cause severe disease in humans.

Fifty percent (50%) of isolates did not carry any *nle*-encoding genes. Isolates which did not posess any *nle*-encoding genes belonged to serotypes that have been incriminated in mild or uncomplicated diarrhea (STEC O26:H2, STEC O26:H8, STEC O26:H21, STEC O121:H8), hemorrhagic colitis (STEC O26:H7, STEC O145:H7) and HUS (STEC O26:H11) in humans^59,60^, and serotypes that have never been associated with human illness^2,78,79^. The lack of *nle*-encoding genes in STEC serotypes that have been previously implicated in mild to severe disease in humans, suggests that the capacity of these strains to cause disease in humans may not be dependent upon currently known non-LEE effectors. However, the absence of known non-LEE effector genes in STEC isolates that have never been implicated in human disease may also explain why these isolates have never been incriminated in human disease.

Antimicrobial resistance profiling showed that almost all (97.9%) the STEC isolates were susceptible to all the 15 tested antimicrobials except for three STEC isolates that were antimicrobial resistant. The three resistant isolates belonged to STEC O26:H11 (tetracycline), STEC O26:H4 (tetracycline and ampicillin) and STEC O45:H21 (amoxicillin-clavulanic acid and cephalothin). Similar findings were made by Dong *et al*.^49^, who also reported resistance to ampicillin and tetracycline among cattle STEC isolates. However, higher antimicrobial resistance levels to tetracycline, ampicillin, cephalothin and amoxicillin-clavulanic acid have been previously reported in a number of studies in STEC isolates^35,36,80^. The very low antimicrobial resistance rates observed in this study suggest that the selection pressure exerted on cattle farms from which the STEC isolates were recovered is negligible. Cattle on cow-calf operations in South Africa graze on pastureland all year round and are not supplemented with feed containing antimicrobial promoters that usually exert selective pressure on intestinal flora such as STEC and facilitate proliferation and development of antimicrobial resistant strains.

PFGE revealed that the STEC isolates under study were highly diverse and only a few isolates had identical fingerprints in individual serogroups. Isolates with identical fingerprints either belonged to the same serotype or were recovered from the same animal or farm. The high diversity observed among the STEC isolates under study is reflection of the high genetic flow occurring among STEC isolates through gene acquisition, shuffling and loss, particularly genes that are encoded on mobile genetic elements including plasmids, bacteriophages and pathogenicity islands.

In conclusion, the majority of STEC isolates were *stx1*, *stx2a, stx2c* and *stx2d* positive but lacked *eaeA*. Plasmid-encoded genes (*hlyA, saa, subA* and *espP)* were detected in most of the isolates but *katP* and *etpD* genes were only observed in a very small number of isolates that were also *eaeA*-positive. A complete plasmid, (*ehxA, etpD, katP* and *esp*P) was observed in STEC O157:H7 isolates mainly. O island and *nle* marker genes were absent in most isolates, except for OI-43/48-associated genes (*terC* and *iha*), which were prevalent in more than 80% of isolates. STEC O157:H7, STEC O145:H28 and STEC O103:H2 and some STEC O26:H2 isolates possessed the highest number of virulence-associated genes. These serotypes which are frequently implicated in severe STEC disease in humans carried *nle* marker genes, such as *nleB*, *nleE*, *ent/espL2*, *nleG2-3, nleG5-2*, *nleG9*, *nleG2-1* and *nleB2*, which are considered a “hallmark” of highly virulent STEC strains^19^.

To our knowledge, this is the first detailed characterization of a large number of cattle STEC isolates from South Africa. This study provides much needed data on the molecular characteristics of STEC serotypes from beef cattle in South Africa. Further studies using whole-genome sequencing (WGS) will be needed to fully assess the virulence potential of these cattle STEC isolates for humans.

## Supplementary information

SUPPLEMENTARY INFO
