# Supplementary material for: Molecular profiling and antimicrobial resistance of Shiga toxin-producing Escherichia coli O26, O45, O103, O121, O145 and O157 isolates from cattle on cow-calf operations in South Africa
Source: Sci Rep. 2019 Aug 15;9:11930. doi: 10.1038/s41598-019-47948-1 (PMC6695430; doi:10.1038/s41598-019-47948-1)
Supplement: Supplementary file 1 — SUPPLEMENTARY INFO [file 41598_2019_47948_MOESM1_ESM.docx]

**Supplementary Information for Scientific Reports**

**Molecular profiling and antimicrobial resistance of Shiga toxin-producing *Escherichia coli* O26, O45, O103, O121, O145 and O157 isolates from cattle on cow-calf operations in South Africa.**

**Musafiri Karama^1*^, Alfred O. Mainga^1^, Beniamino T. Cenci Goga^1,2^, Mogau Malahlela^1^, Saeed El-Ashram^3,4^, Alan Kalake^3^.**

**^1^Veterinary Public Health Section, Department of Paraclinical Sciences, Faculty of Veterinary Science, University of Pretoria, Onderstepoort, South Africa;** [**musafiri.karama@up.ac.za**](mailto:musafiri.karama@up.ac.za)**, Tel.: +27 529 8181/8408.**

**^2^Dipartimento di Scienze Biopatologiche, Laboratorio di Ispezione degli Alimenti di Origine Animale, Facoltà di Medicina Veterinaria, Università degli Studi di Perugia, Perugia, Italy;** [**beniamino.cencigoga@unipg.it**](mailto:beniamino.cencigoga@unipg.it)

**^3^School of Life Science and Engineering, Foshan University, Foshan Guangdong 528231; saeed_elashram@yahoo.com**

**^4^Faculty of Science, Kafrelsheikh University, Egypt**

**^5^Gauteng Department of Agriculture and Rural Development (GDARD), Johannesburg, South Africa;** [**alan.kalake@gauteng.gov.za**](mailto:alan.kalake@gauteng.gov.za)

**Table S1. Distribution of Plasmid encoded and PAI Genes and Markers in *eaeA*-positive serotypes**

| **STEC**  **Serotype** | **stx Variants** | *eaeA* | **Plasmid encoded Genes** | | | | **OI-122** | | | | | **OI-57** | | | **OI-71** | | | | | | **OI-36** | | | | **OI-43/48** | | |
| --- | --- | --- | --- | --- | --- | --- | --- | --- | --- | --- | --- | --- | --- | --- | --- | --- | --- | --- | --- | --- | --- | --- | --- | --- | --- | --- | --- |
|  |  |  | *ehxA* | *katP* | *espP* | *etpD* | *pagC* | *sen-(Z4326)* | *ent/espL2* | *nleB & E* | *efa1 (Z4332/Z4333)* | *nleG 2-3* | *nleG 6-2* | *nleG 5-2* | *nleG9* | *nleG* | *nleF* | *nleH 1-2* | *nleA* | *nleG 2-1* | *nleB2* | *nleC* | *nleD* | *nleH 1-1* | ***terC*** | ***ureC*** | ***iha*** |
| **O26:H2 (2)** | ***stx1d*** | **2** | **2** | 0 | 2 | 0 | 2 | 2 | 1 | 2 | 0 | 2 | 0 | 1 | 0 | 0 | 0 | 0 | 2 | 2 | 2 | 1 | 1 | 0 | 2 | 2 | 2 |
| **O103:H2 (1)** | ***stx1*** | **1** | **1** | 0 | 0 | 1 | 1 | 1 | 1 | 1 | 1 | 1 | 0 | 1 | 1 | 1 | 1 | 1 | 0 | 1 | 1 | 0 | 1 | 1 | 1 | 1 | 0 |
| **O145:H28 (2)** | ***stx2, 2c*** | **2** | **2** | 2 | 2 | 0 | 0 | 2 | 2 | 2 | 2 | 2 | 0 | 2 | 2 | 0 | 2 | 0 | 2 | 2 | 2 | 0 | 0 | 0 | 2 | 2 | 2 |
| **O145:H28 (1)** | ***stx2, 2c, 2d*** | **1** | **1** | 1 | 1 | 0 | 0 | 1 | 1 | 1 | 1 | 1 | 1 | 1 | 1 | 0 | 1 | 0 | 1 | 1 | 1 | 0 | 1 | 0 | 1 | 1 | 1 |
| **O145:HNT (1)** | ***stx2, 2c, 2d*** | **1** | **1** | 1 | 1 | 0 | 0 | 1 | 1 | 1 | 0 | 1 | 0 | 1 | 1 | 0 | 1 | 1 | 1 | 1 | 0 | 0 | 1 | 1 | 1 | 1 | 1 |
| **O157:H7 (7)** | ***stx2, 2c, 2d*** | **7** | **7** | 7 | 7 | 7 | 7 | 7 | 7 | 7 | 7 | 7 | 7 | 7 | 7 | 1 | 0 | 7 | 7 | 7 | 7 | 7 | 7 | 7 | 7 | 7 | 7 |
| **O157:H7 (2)** | ***stx2, 2c, 2d*** | **2** | **2** | 2 | 2 | 2 | 2 | 2 | 2 | 2 | 2 | 2 | 2 | 2 | 2 | 0 | 0 | 2 | 2 | 2 | 2 | 2 | 2 | 2 | 2 | 2 | 2 |
| **O157:H28 (1)** | ***stx2, 2c*** | **1** | **1** | 1 | 1 | 0 | 0 | 1 | 1 | 1 | 1 | 1 | 0 | 1 | 1 | 0 | 1 | 0 | 1 | 1 | 1 | 1 | 0 | 0 | 1 | 1 | 1 |
|  | **Total** | 17 | 17 | 14 | 16 | 10 | 12 | 17 | 16 | 17 | 14 | 17 | 10 | 16 | 15 | 2 | 6 | 11 | 16 | 17 | 16 | 11 | 14 | 11 | 17 | 17 | 16 |
|  |  |  |  |  |  |  | ***pagC*** | ***sen*** | ***Ent/espL2*** | ***nleB&E*** | ***efa*** | ***nleG2-3*** | ***Nleg6-2*** | ***nleG5-2*** | ***nleG9*** | ***nleG*** | ***nleF*** | ***nleH1-2*** | ***nleA*** | ***nleG2-1*** | ***nleB2*** | ***nleC*** | ***nleD*** | ***nleH1-1*** | ***terC*** | ***ureC*** | ***iha*** |
|  | **% Positive** | **100** | **100** | **82,4** | **94,1** | **58,8** | **70,6** | **100** | **94,1** | **100** | **82,4** | **100** | **64,7** | **94,1** | **88,2** | **11,8** | **35,3** | **64,7** | **94,1** | **100** | **94,1** | **64,7** | **82,4** | **64,7** | **100** | **100** | **94,1** |

**Table S2. Distribution of Pathogenicity - and Plasmid- encoded Genes and Markers**

|  |  |  | **Plasmid encoded Genes** | | | | | | **OI-122** | | | | | | | **OI-57** | | | **OI-71** | | | | | | **OI-36** | | | | | | | **OI-43/48** | | |  |
| --- | --- | --- | --- | --- | --- | --- | --- | --- | --- | --- | --- | --- | --- | --- | --- | --- | --- | --- | --- | --- | --- | --- | --- | --- | --- | --- | --- | --- | --- | --- | --- | --- | --- | --- | --- |
| **SEROTYPE** | **No.of Isolates** | ***eaeA*** | ***hlyA*** | ***saa*** | ***subA*** | ***katP*** | ***espP*** | ***etpD*** | ***pagC*** | ***sen (Z4326)*** | ***ent/ espL2*** | ***efa1 (Z4332)*** | ***efa1 (Z4333)*** | ***nleB*** | ***nleE*** | ***nleG2-3*** | ***nleG6-2*** | ***nleG5-2*** | ***nleG9*** | ***nleG*** | ***nleF*** | ***nleH1-2*** | ***nleA*** | ***nleG2-1*** | ***nleB2*** | | ***nleC*** | ***nleD*** | | ***nleH1-1*** | | ***ter-C*** | ***ureC*** | ***iha*** |  |
| **O26:H2** | **7** | **-** | **+** | **+** | **-** | **-** | **+** | **-** | **+** | **-** | **-** | **-** | **-** | **-** | **-** | **-** | **-** | **-** | **-** | **-** | **-** | **-** | **-** | **-** | **-** | | **-** | **-** | | **-** | | **+** | **-** | **+** |  |
| **O26:H2** | **1** | **-** | **+** | **+** | **-** | **-** | **-** | **-** | **+** | **-** | **-** | **-** | **-** | **-** | **-** | **-** | **-** | **-** | **-** | **-** | **-** | **-** | **-** | **-** | **-** | | **-** | **-** | | **-** | | **+** | **-** | **-** |  |
| **O26:H2** | **1** | **-** | **-** | **-** | **-** | **-** | **+** | **-** | **+** | **-** | **-** | **-** | **-** | **-** | **-** | **-** | **-** | **-** | **-** | **-** | **-** | **-** | **-** | **-** | **-** | | **-** | **-** | | **-** | | **+** | **-** | **+** |  |
| **O26:H2** | **1** | **-** | **+** | **+** | **-** | **-** | **+** | **-** | **+** | **-** | **-** | **-** | **-** | **-** | **-** | **-** | **-** | **-** | **-** | **-** | **-** | **-** | **-** | **-** | **-** | | **-** | **-** | | **-** | | **+** | **+** | **+** |  |
| **O26:H2** | **1** | **-** | **+** | **+** | **-** | **-** | **+** | **-** | **+** | **-** | **-** | **-** | **-** | **-** | **-** | **-** | **-** | **-** | **-** | **-** | **-** | **-** | **-** | **-** | **-** | | **-** | **-** | | **-** | | **+** | **+** | **-** |  |
| **O26:H2** | **6** | **-** | **+** | **+** | **-** | **-** | **+** | **-** | **+** | **-** | **-** | **-** | **-** | **-** | **-** | **-** | **-** | **-** | **-** | **-** | **-** | **-** | **-** | **-** | **-** | | **-** | **-** | | **-** | | **+** | **-** | **+** |  |
| **O26:H2** | **1** | **+** | **+** | **-** | **-** | **-** | **+** | **-** | **+** | **+** | **-** | **-** | **-** | **+** | **+** | **+** | **-** | **-** | **-** | **-** | **-** | **-** | **+** | **+** | **+** | | **+** | **-** | | **-** | | **+** | **+** | **+** |  |
| **O26:H2** | **1** | **+** | **+** | **-** | **-** | **-** | **+** | **-** | **+** | **+** | **+** | **-** | **-** | **+** | **+** | **+** | **-** | **+** | **-** | **-** | **-** | **-** | **+** | **+** | **+** | | **-** | **+** | | **-** | | **+** | **+** | **+** |  |
| **O26:H2** | **1** | **-** | **+** | **+** | **-** | **-** | **+** | **-** | **+** | **+** | **+** | **-** | **+** | **-** | **+** | **+** | **+** | **+** | **+** | **-** | **-** | **+** | **-** | **+** | **+** | | **-** | **+** | | **+** | | **+** | **+** | **+** |  |
| O26:H4 | **1** | **-** | **-** | **+** | **-** | **-** | **-** | **-** | **-** | **-** | **-** | **-** | **-** | **-** | **-** | **-** | **-** | **-** | **-** | **-** | **-** | **+** | **-** | **-** | **-** | | **-** | **+** | | **+** | | **-** | **+** | **-** |  |
| **O26:H7** | **1** | **-** | **+** | **+** | **-** | **-** | **-** | **-** | **-** | **-** | **-** | **-** | **-** | **-** | **-** | **-** | **-** | **-** | **-** | **-** | **-** | **-** | **-** | **-** | **-** | | **-** | **-** | | **-** | | **+** | **+** | **+** |  |
| **O26:H7** | **1** | **-** | **+** | **+** | **+** | **-** | **+** | **-** | **-** | **-** | **-** | **-** | **-** | **-** | **-** | **-** | **-** | **-** | **-** | **-** | **-** | **-** | **-** | **-** | **-** | | **-** | **-** | | **-** | | **+** | **+** | **+** |  |
| **O26:H7** | **1** | **-** | **-** | **-** | **-** | **-** | **-** | **-** | **-** | **-** | **-** | **-** | **-** | **-** | **-** | **-** | **-** | **-** | **+** | **-** | **-** | **+** | **-** | **-** | **-** | | **-** | **-** | | **+** | | **-** | **-** | **-** |  |
| **O26:H8** | **1** | **-** | **+** | **+** | **-** | **-** | **+** | **-** | **+** | **-** | **-** | **-** | **-** | **-** | **-** | **-** | **-** | **-** | **-** | **-** | **-** | **-** | **-** | **-** | **-** | | **-** | **-** | | **-** | | **-** | **-** | **+** |  |
| **O26:H8** | **1** | **-** | **+** | **+** | **-** | **-** | **-** | **-** | **+** | **-** | **-** | **+** | **+** | **-** | **-** | **-** | **-** | **-** | **-** | **-** | **-** | **-** | **-** | **-** | **-** | | **-** | **-** | | **-** | | **+** | **+** | **+** |  |
| **O26:H8** | **1** | **-** | **+** | **+** | **-** | **-** | **-** | **-** | **+** | **-** | **-** | **-** | **-** | **-** | **-** | **-** | **-** | **-** | **-** | **-** | **-** | **-** | **-** | **-** | **-** | | **-** | **-** | | **-** | | **-** | **-** | **+** |  |
| **O26:H8** | **1** | **-** | **+** | **+** | **-** | **-** | **-** | **-** | **+** | **-** | **-** | **-** | **-** | **-** | **-** | **-** | **-** | **-** | **-** | **-** | **-** | **-** | **-** | **-** | **-** | | **-** | **-** | | **-** | | **-** | **-** | **+** |  |
| **O26:H8** | **1** | **-** | **+** | **+** | **-** | **-** | **-** | **-** | **+** | **-** | **-** | **-** | **-** | **-** | **-** | **-** | **-** | **-** | **-** | **-** | **-** | **-** | **-** | **-** | **-** | | **-** | **-** | | **-** | | **-** | **-** | **+** |  |
| **O26:H8** | **3** | **-** | **+** | **+** | **-** | **-** | **+** | **-** | **+** | **-** | **-** | **-** | **-** | **-** | **-** | **-** | **-** | **-** | **-** | **-** | **-** | **-** | **-** | **-** | **-** | | **-** | **-** | | **-** | | **-** | **-** | **+** |  |
| O26:H11 | **1** | **-** | **-** | **+** | **+** | **-** | **-** | **-** | **+** | **-** | **-** | **-** | **-** | **-** | **-** | **-** | **-** | **-** | **-** | **-** | **-** | **-** | **-** | **-** | **-** | | **-** | **-** | | **-** | | **+** | **-** | **+** |  |
| O26:H11 | **1** | **-** | **+** | **+** | **+** | **-** | **+** | **-** | **+** | **-** | **-** | **-** | **-** | **-** | **-** | **-** | **-** | **-** | **-** | **-** | **-** | **-** | **-** | **-** | **-** | | **-** | **-** | | **-** | | **-** | **-** | **+** |  |
| O26:H11 | **1** | **-** | **+** | **+** | **+** | **-** | **+** | **-** | **+** | **-** | **-** | **-** | **-** | **-** | **-** | **-** | **-** | **-** | **-** | **-** | **-** | **-** | **-** | **-** | **-** | | **-** | **-** | | **-** | | **-** | **-** | **+** |  |
| O26:H16 | **1** | **-** | **-** | **-** | **-** | **-** | **-** | **-** | **+** | **-** | **-** | **-** | **-** | **-** | **-** | **-** | **-** | **-** | **+** | **-** | **-** | **-** | **-** | **-** | **-** | | **-** | **-** | | **-** | | **-** | **-** | **+** |  |
| O26:H16 | **1** | **-** | **+** | **+** | **-** | **-** | **+** | **-** | **-** | **-** | **-** | **-** | **-** | **-** | **-** | **-** | **-** | **-** | **+** | **-** | **-** | **+** | **-** | **-** | **-** | | **-** | **+** | | **+** | | **-** | **-** | **+** |  |
| O26:H19 | **1** | **-** | **+** | **+** | **-** | **-** | **-** | **-** | **-** | **-** | **-** | **-** | **-** | **-** | **-** | **-** | **-** | **-** | **-** | **-** | **-** | **-** | **-** | **-** | **-** | | **-** | **-** | | **-** | | **­-** | **-** | **+** |  |
| O26:H19 | **1** | **-** | **+** | **+** | **+** | **-** | **+** | **-** | **-** | **-** | **-** | **-** | **-** | **-** | **-** | **-** | **-** | **-** | **-** | **-** | **-** | **-** | **-** | **-** | **-** | | **-** | **-** | | **-** | | **-** | **-** | **+** |  |
| Total (26) |  |  |  |  |  |  |  |  |  |  |  |  |  |  |  |  |  |  |  |  |  |  |  |  |  | |  |  | |  | |  |  |  |  |
|  |  |  | **Plasmid encoded Genes** | | | | | | **OI-122** | | | | | | | **OI-57** | | | **OI-71** | | | | | | **OI-36** | | | | | | | **OI-43/48** | | |  |
| **SEROTYPE** | **No.of Isolate** | ***eaeA*** | ***hlyA*** | ***saa*** | ***subA*** | ***katP*** | ***espP*** | ***etpD*** | ***pagC*** | ***sen (Z4326)*** | ***ent/ espL2*** | ***efa1 (Z4332)*** | ***efa1 (Z4333)*** | ***nleB*** | ***nleE*** | ***nleG2-3*** | ***nleG6-2*** | ***nleG5-2*** | ***nleG9*** | ***nleG*** | ***nleF*** | ***nleH1-2*** | ***nleA*** | ***nleG2-1*** | ***nleB2*** | | ***nleC*** | ***nleD*** | | ***nleH1-1*** | | ***ter-C*** | ***ureC*** | ***iha*** |  |
| **O26:H21** | **1** | **-** | **+** | **+** | **+** | **-** | **+** | **-** | **+** | **-** | **-** | **-** | **-** | **-** | **-** | **-** | **-** | **-** | **-** | **-** | **-** | **-** | **-** | **-** | **-** | | **-** | **-** | | **-** | | **-** | **-** | **+** |  |
| **O26:H21** | **1** | **-** | **+** | **+** | **+** | **-** | **+** | **-** | **+** | **-** | **-** | **-** | **-** | **-** | **-** | **-** | **-** | **-** | **-** | **-** | **-** | **-** | **-** | **-** | **-** | | **-** | **-** | | **-** | | **-** | **-** | **+** |  |
| **O26:H21** | **1** | **-** | **-** | **-** | **-** | **-** | **-** | **-** | **-** | **+** | **+** | **-** | **+** | **+** | **+** | **+** | **+** | **+** | **-** | **+** | **+** | **-** | **+** | **-** | **+** | | **-** | **-** | | **-** | | **-** | **+** | **+** |  |
| **O26:H21** | **2** | **-** | **-** | **+** | **+** | **-** | **-** | **-** | **-** | **-** | **-** | **-** | **-** | **-** | **-** | **-** | **-** | **-** | **-** | **-** | **-** | **-** | **-** | **-** | **-** | | **-** | **-** | | **-** | | **+** | **-** | **+** |  |
| **O26:H21** | **1** | **-** | **+** | **+** | **-** | **-** | **+** | **-** | **-** | **-** | **-** | **-** | **-** | **-** | **-** | **-** | **-** | **-** | **-** | **-** | **-** | **-** | **-** | **-** | **-** | | **-** | **-** | | **-** | | **-** | **+** | **+** |  |
| **O26:H21** | **1** | **-** | **+** | **+** | **-** | **-** | **+** | **-** | **+** | **-** | **-** | **-** | **-** | **-** | **-** | **-** | **-** | **-** | **-** | **-** | **-** | **-** | **-** | **-** | **-** | | **-** | **-** | | **-** | | **+** | **+** | **+** |  |
| O26:H28 | **1** | **-** | **-** | **-** | **-** | **-** | **-** | **-** | **-** | **-** | **-** | **-** | **-** | **-** | **-** | **-** | **-** | **-** | **+** | **-** | **-** | **-** | **-** | **-** | **-** | | **-** | **-** | | **-** | | **-** | **-** | **-** |  |
| O26:H28 | **1** | **-** | **+** | **+** | **-** | **-** | **+** | **-** | **+** | **-** | **-** | **-** | **-** | **-** | **-** | **-** | **-** | **-** | **+** | **-** | **-** | **-** | **-** | **-** | **-** | | **-** | **-** | | **-** | | **-** | **-** | **+** |  |
| O26:H38 | **2** | **-** | **+** | **+** | **-** | **-** | **+** | **-** | **-** | **-** | **-** | **-** | **-** | **-** | **-** | **-** | **-** | **-** | **-** | **-** | **-** | **-** | **-** | **-** | **-** | | **-** | **-** | | **-** | | **-** | **-** | **+** |  |
| O26:H45 | **1** | **-** | **+** | **+** | **-** | **-** | **-** | **-** | **-** | **-** | **-** | **-** | **-** | **-** | **-** | **-** | **-** | **-** | **-** | **-** | **-** | **-** | **-** | **-** | **-** | | **-** | **-** | | **-** | | **-** | **-** | **-** |  |
| O26:HNT | **1** | **-** | **+** | **+** | **-** | **-** | **-** | **-** | **-** | **-** | **-** | **-** | **-** | **-** | **-** | **-** | **-** | **-** | **-** | **-** | **-** | **-** | **-** | **-** | **-** | | **-** | **-** | | **-** | | **-** | **-** | **+** |  |
| O26:HNT | **1** | **-** | **+** | **+** | **+** | **-** | **-** | **-** | **+** | **-** | **-** | **-** | **-** | **-** | **-** | **-** | **-** | **-** | **-** | **-** | **-** | **-** | **-** | **-** | **-** | | **-** | **-** | | **-** | | **-** | **+** | **+** |  |
| O26:HNT | **2** | **-** | **+** | **+** | **+** | **-** | **-** | **-** | **+** | **-** | **-** | **-** | **-** | **-** | **-** | **-** | **-** | **-** | **-** | **-** | **-** | **-** | **-** | **-** | **-** | | **+** | **-** | | **-** | | **+** | **+** | **+** |  |
| **O157:H7** | **1** | **+** | **+** | **-** | **-** | **+** | **+** | **+** | **+** | **+** | **+** | **+** | **+** | **+** | **+** | **+** | **+** | **+** | **+** | **-** | **-** | **+** | **+** | **+** | **+** | | **+** | **+** | | **+** | | **+** | **+** | **+** |  |
| **O157:H7** | **1** | **+** | **+** | **-** | **-** | **+** | **+** | **+** | **+** | **+** | **+** | **+** | **+** | **+** | **+** | **+** | **+** | **+** | **+** | **-** | **-** | **+** | **+** | **+** | **+** | | **+** | **+** | | **+** | | **+** | **+** | **+** |  |
| O157:H2 | **1** | **-** | **+** | **+** | **-** | **-** | **+** | **-** | **+** | **-** | **-** | **-** | **-** | **-** | **-** | **-** | **-** | **-** | **-** | **-** | **-** | **-** | **-** | **-** | **-** | | **-** | **-** | | **-** | | **+** | **+** | **+** |  |
| **O157:H7** | **6** | **+** | **+** | **-** | **-** | **+** | **+** | **+** | **+** | **+** | **+** | **+** | **+** | **+** | **+** | **+** | **+** | **+** | **+** | **-** | **-** | **+** | **+** | **+** | **+** | | **+** | **+** | | **+** | | **+** | **+** | **+** |  |
| **O157:H7** | **1** | **+** | **+** | **-** | **-** | **+** | **+** | **+** | **+** | **+** | **+** | **+** | **+** | **+** | **+** | **+** | **+** | **+** | **+** | **+** | **-** | **+** | **+** | **+** | **+** | | **+** | **+** | | **+** | | **+** | **+** | **+** |  |
| O157:H19 | **1** | **-** | **+** | **+** | **-** | **-** | **+** | **-** | **-** | **+** | **+** | **-** | **-** | **+** | **-** | **-** | **+** | **+** | **-** | **-** | **-** | **-** | **-** | **+** | **+** | | **+** | **+** | | **+** | | **+** | **+** | **+** |  |
| O157:H28 | **1** | **+** | **+** | **-** | **-** | **+** | **+** | **-** | **-** | **+** | **+** | **+** | **+** | **+** | **+** | **+** | **-** | **+** | **+** | **-** | **+** | **-** | **+** | **+** | **+** | | **+** | **-** | | **-** | | **+** | **+** | **+** |  |
| O145:H2 | **1** | **-** | **+** | **+** | **-** | **-** | **+** | **-** | **+** | **-** | **-** | **-** | **-** | **-** | **-** | **-** | **-** | **-** | **-** | **-** | **-** | **-** | **-** | **-** | **-** | | **-** | **-** | | **-** | | **+** | **-** | **+** |  |
| **O145:H7** | **1** | **-** | **+** | **+** | **-** | **-** | **-** | **-** | **-** | **-** | **-** | **-** | **-** | **-** | **-** | **-** | **-** | **-** | **-** | **-** | **-** | **-** | **-** | **-** | **-** | | **-** | **-** | | **-** | | **+** | **+** | **+** |  |
| **O145:H8** | **1** | **-** | **+** | **+** | **-** | **-** | **+** | **-** | **+** | **-** | **-** | **-** | **-** | **-** | **-** | **-** | **-** | **-** | **-** | **-** | **-** | **-** | **-** | **-** | **-** | | **-** | **-** | | **-** | | **+** | **-** | **+** |  |
| O145:H11 | **1** | **-** | **+** | **+** | **+** | **-** | **+** | **-** | **+** | **-** | **-** | **-** | **-** | **-** | **-** | **-** | **-** | **-** | **-** | **-** | **-** | **-** | **-** | **-** | **-** | | **-** | **-** | | **-** | | **+** | **-** | **+** |  |
| O145:H19 | **2** | **-** | **+** | **+** | **-** | **-** | **+** | **-** | **-** | **-** | **-** | **-** | **-** | **-** | **-** | **-** | **-** | **-** | **-** | **-** | **-** | **-** | **-** | **-** | **-** | | **-** | **-** | | **-** | | **+** | **+** | **+** |  |
| O145:H19 | **1** | **-** | **+** | **+** | **-** | **-** | **+** | **-** | **-** | **-** | **-** | **-** | **-** | **-** | **-** | **-** | **-** | **-** | **-** | **-** | **-** | **-** | **-** | **-** | **-** | | **-** | **-** | | **-** | | **+** | **-** | **+** |  |
| O145:H19 | **4** | **-** | **+** | **+** | **-** | **-** | **+** | **-** | **-** | **-** | **-** | **-** | **-** | **-** | **-** | **-** | **-** | **+** | **-** | **-** | **-** | **-** | **-** | **-** | **-** | | **-** | **-** | | **-** | | **+** | **+** | **+** |  |
| O145:H19 | **1** | **-** | **+** | **+** | **-** | **-** | **+** | **-** | **-** | **-** | **-** | **-** | **-** | **-** | **-** | **-** | **-** | **-** | **-** | **-** | **-** | **-** | **-** | **-** | **-** | | **-** | **-** | | **-** | | **+** | **+** | **+** |  |
| O145:H19 | **1** | **-** | **+** | **+** | **-** | **-** | **+** | **-** | **-** | **-** | **-** | **-** | **-** | **-** | **-** | **-** | **-** | **-** | **-** | **-** | **-** | **-** | **-** | **-** | **-** | | **-** | **-** | | **-** | | **+** | **-** | **+** |  |
| Total (29) |  |  |  |  |  |  |  |  |  |  |  |  |  |  |  |  |  |  |  |  |  |  |  |  |  | |  |  | |  | |  |  |  |  |
|  |  |  | **Plasmid encoded Genes** | | | | | | **OI-122** | | | | | | | **OI-57** | | | **OI-71** | | | | | | **OI-36** | | | | | | | **OI-43/48** | | |  |
| **SEROTYPE** | **No.of Isolate** | ***eaeA*** | ***hlyA*** | ***saa*** | ***subA*** | ***katP*** | ***espP*** | ***etpD*** | ***pagC*** | ***sen (Z4326)*** | ***ent/ espL2*** | ***efa1 (Z4332)*** | ***efa1 (Z4333)*** | ***nleB*** | ***nleE*** | ***nleG2-3*** | ***nleG6-2*** | ***nleG5-2*** | ***nleG9*** | ***nleG*** | ***nleF*** | ***nleH1-2*** | ***nleA*** | ***nleG2-1*** | ***nleB2*** | | ***nleC*** | ***nleD*** | | ***nleH1-1*** | | ***ter-C*** | ***ureC*** | ***iha*** |  |
| O145:H19 | **2** | **-** | **+** | **+** | **-** | **-** | **+** | **-** | **-** | **-** | **-** | **-** | **-** | **-** | **-** | **+** | **+** | **+** | **-** | **-** | **-** | **+** | **-** | **-** | **-** | | **+** | **+** | | **+** | | **+** | **+** | **+** |  |
| O145:H19 | **1** | **-** | **+** | **+** | **-** | **-** | **+** | **-** | **-** | **-** | **-** | **-** | **-** | **-** | **-** | **+** | **+** | **+** | **-** | **-** | **-** | **+** | **-** | **-** | **-** | | **-** | **+** | | **+** | | **+** | **+** | **+** |  |
| O145:H19 | **1** | **-** | **+** | **+** | **+** | **-** | **+** | **-** | **-** | **-** | **-** | **-** | **-** | **-** | **-** | **-** | **-** | **-** | **-** | **-** | **-** | **-** | **-** | **-** | **-** | | **-** | **-** | | **-** | | **+** | **+** | **+** |  |
| **O145:H28** | **2** | **+** | **+** | **-** | **-** |  | **+** | **-** | **-** | **+** | **+** | **+** | **+** | **+** | **+** | **+** | **-** | **+** | **+** | **-** | **+** | **-** | **+** | **+** | **+** | | **-** | **-** | | **-** | | **+** | **+** | **+** |  |
| **O145:H28** | **1** | **+** | **+** | **-** | **-** |  | **+** | **-** | **-** | **+** | **+** | **+** | **+** | **+** | **+** | **+** | **+** | **+** | **+** | **-** | **+** | **-** | **+** | **+** | **+** | | **-** | **+** | | **-** | | **+** | **+** | **+** |  |
| O145:HNT | **1** | **+** | **+** | **-** | **-** | **+** | **+** | **-** | **-** | **+** | **+** | **-** | **-** | **+** | **+** | **+** | **-** | **+** | **+** | **-** | **+** | **+** | **+** | **+** | **-** | | **-** | **+** | | **+** | | **+** | **+** | **+** |  |
| O145:HNT | **1** | **-** | **+** | **+** | **+** | **-** | **+** | **-** | **-** | **-** | **-** | **-** | **-** | **-** | **-** | **-** | **-** | **-** | **-** | **-** | **-** | **-** | **-** | **-** | **-** | | **-** | **-** | | **-** | | **+** | **+** | **+** |  |
| O145:HNT | **1** | **-** | **+** | **+** | **-** | **-** | **+** | **-** | **-** | **-** | **-** | **-** | **-** | **-** | **-** | **-** | **-** | **-** | **-** | **-** | **-** | **-** | **-** | **-** | **-** | | **-** | **-** | | **-** | | **+** | **-** | **+** |  |
| **O121:H8** | **3** | **-** | **+** | **+** | **+** | **-** | **+** | **-** | **+** | **-** | **-** | **-** | **-** | **-** | **-** | **-** | **-** | **-** | **-** | **-** | **-** | **-** | **-** | **-** | **-** | | **-** | **-** | | **-** | | **+** | **-** | **+** |  |
| **O121:H8** | **1** | **-** | **+** | **+** | **+** | **-** | **-** | **-** | **+** | **-** | **-** | **-** | **-** | **-** | **-** | **-** | **-** | **-** | **-** | **-** | **-** | **-** | **-** | **-** | **-** | | **-** | **-** | | **-** | | **+** | **-** | **-** |  |
| **O121:H8** | **1** | **-** | **+** | **+** | **+** | **-** | **-** | **-** | **+** | **-** | **-** | **-** | **-** | **-** | **-** | **-** | **-** | **-** | **-** | **-** | **-** | **-** | **-** | **-** | **-** | | **-** | **-** | | **-** | | **+** | **-** | **+** |  |
| **O121:H8** | **1** | **-** | **+** | **+** | **+** | **-** | **-** | **-** | **+** | **-** | **-** | **-** | **-** | **-** | **-** | **-** | **-** | **-** | **-** | **-** | **-** | **-** | **-** | **-** | **-** | | **-** | **-** | | **-** | | **+** | **+** | **+** |  |
| **O121:H8** | **1** | **-** | **+** | **+** | **+** | **-** | **+** | **-** | **-** | **-** | **-** | **-** | **-** | **-** | **-** | **-** | **-** | **-** | **-** | **-** | **-** | **-** | **-** | **-** | **-** | | **-** | **-** | | **-** | | **+** | **+** | **+** |  |
| **O121:H8** | **1** | **-** | **+** | **+** | **+** | **-** | **-** | **-** | **+** | **-** | **-** | **-** | **-** | **-** | **-** | **-** | **-** | **-** | **-** | **-** | **-** | **-** | **-** | **-** | **-** | | **-** | **-** | | **-** | | **+** | **-** | **+** |  |
| O121:H21 | **1** | **-** | **+** | **+** | **+** | **-** | **-** | **-** | **-** | **-** | **-** | **-** | **-** | **-** | **-** | **-** | **-** | **-** | **-** | **-** | **-** | **-** | **-** | **-** | **-** | | **-** | **-** | | **-** | | **-** | **+** | **+** |  |
| O121:HNT | **1** | **-** | **+** | **+** | **+** | **-** | **+** | **-** | **+** | **-** | **-** | **-** | **-** | **-** | **-** | **-** | **-** | **-** | **-** | **-** | **-** | **-** | **-** | **-** | **-** | | **-** | **-** | | **-** | | **+** | **-** | **+** |  |
| **O103:H2** | **1** | **+** | **+** | **-** | **-** | **-** | **-** | **+** | **+** | **+** | **+** | **+** | **+** | **+** | **+** | **+** | **-** | **+** | **+** | **+** | **+** | **+** | **-** | **+** | **+** | | **-** | **+** | | **+** | | **+** | **+** | **-** |  |
| **O103:H21** | **1** | **-** | **-** | **-** | **-** | **-** | **-** | **+** | **+** | **-** | **-** | **-** | **-** | **-** | **-** | **-** | **-** | **+** | **-** | **-** | **-** | **-** | **-** | **-** | **-** | | **-** | **-** | | **-** | | **-** | **-** | **-** |  |
| **O45:H2** | **1** | **-** | **+** | **+** | **-** | **-** | **+** | **-** | **+** | **+** | **+** | **-** | **+** | **-** | **+** | **+** | **+** | **-** | **-** | **-** | **-** | **+** | **-** | **-** | **-** | | **-** | **-** | | **+** | | **-** | **+** | **+** |  |
| O45:H8 | **1** | **-** | **+** | **+** | **-** | **-** | **+** | **-** | **+** | **+** | **+** | **-** | **-** | **-** | **-** | **-** | **+** | **-** | **-** | **-** | **-** | **-** | **-** | **-** | **-** | | **-** | **-** | | **-** | | **+** | **+** | **+** |  |
| O45:H8 | **1** | **-** | **+** | **+** | **+** | **-** | **+** | **-** | **-** | **+** | **+** | **-** | **-** | **+** | **-** | **+** | **+** | **-** | **-** | **-** | **-** | **-** | **-** | **-** | **-** | | **-** | **-** | | **-** | | **+** | **+** | **+** |  |
| O45:H8 | **1** | **-** | **+** | **+** | **+** | **-** | **+** | **-** | **-** | **+** | **-** | **-** | **-** | **+** | **-** | **-** | **+** | **-** | **-** | **-** | **-** | **-** | **-** | **-** | **-** | | **-** | **-** | | **-** | | **+** | **+** | **+** |  |
| O45:H11 | **1** | **-** | **+** | **+** | **+** | **-** | **+** | **-** | **+** | **+** | **+** | **-** | **+** | **+** | **+** | **+** | **+** | **-** | **-** | **-** | **-** | **+** | **-** | **+** | **+** | | **+** | **+** | | **+** | | **+** | **+** | **+** |  |
| O45:H11 | **3** | **-** | **+** | **+** | **+** | **-** | **+** | **-** | **+** | **+** | **+** | **-** | **+** | **+** | **+** | **+** | **+** | **-** | **-** | **-** | **-** | **+** | **-** | **-** | **-** | | **-** | **+** | | **+** | | **+** | **+** | **+** |  |
| O45:H11 | **2** | **-** | **+** | **+** | **+** | **-** | **+** | **-** | **+** | **+** | **+** | **-** | **+** | **+** | **+** | **+** | **+** | **-** | **-** | **-** | **-** | **+** | **-** | **-** | **-** | | **-** | **-** | | **+** | | **+** | **+** | **+** |  |
| O45:H11 | **2** | **-** | **+** | **+** | **+** | **-** | **+** | **-** | **+** | **+** | **+** | **-** | **+** | **+** | **+** | **+** | **+** | **-** | **+** | **-** | **-** | **+** | **-** | **-** | **-** | | **-** | **+** | | **+** | | **+** | **+** | **+** |  |
| O45:H16 | **1** | **-** | **+** | **+** | **+** | **-** | **+** | **-** | **-** | **+** | **+** | **-** | **+** | **+** | **+** | **+** | **+** | **-** | **-** | **-** | **-** | **+** | **-** | **-** | **+** | | **-** | **+** | | **+** | | **+** | **+** | **+** |  |
| O45:H16 | **1** | **-** | **+** | **+** | **+** | **-** | **+** | **-** | **-** | **+** | **+** | **-** | **-** | **+** | **+** | **+** | **-** | **-** | **-** | **-** | **-** | **-** | **-** | **-** | **-** | | **-** | **-** | | **-** | | **+** | **+** | **+** |  |
| O45:H16 | **1** | **-** | **+** | **+** | **+** | **-** | **+** | **-** | **-** | **-** | **-** | **-** | **-** | **-** | **-** | **-** | **-** | **-** | **-** | **-** | **-** | **-** | **-** | **-** | **-** | | **-** | **-** | | **-** | | **+** | **-** | **+** |  |
| Total (29) |  |  |  |  |  |  |  |  |  |  |  |  |  |  |  |  |  |  |  |  |  |  |  |  |  | |  |  | |  | |  |  |  |  |
|  |  |  | **Plasmid encoded Genes** | | | | | | **OI-122** | | | | | | | **OI-57** | | | **OI-71** | | | | | | **OI-36** | | | | | | | **OI-43/48** | | |  |
| **SEROTYPE** | **No.of Isolate** | ***eaeA*** | ***hlyA*** | ***saa*** | ***subA*** | ***katP*** | ***espP*** | ***etpD*** | ***pagC*** | ***sen (Z4326)*** | ***ent/ espL2*** | ***efa1 (Z4332)*** | ***efa1 (Z4333)*** | ***nleB*** | ***nleE*** | ***nleG2-3*** | ***nleG6-2*** | ***nleG5-2*** | ***nleG9*** | ***nleG*** | ***nleF*** | ***nleH1-2*** | ***nleA*** | ***nleG2-1*** | | ***nleB2*** | | | ***nleC*** | | ***nleD*** | ***nleH1-1*** | ***ter-C*** | ***ureC*** | ***iha*** |
| O45:H19 | **1** | **-** | **+** | **+** | **+** | **-** | **+** | **-** | **-** | **+** | **+** | **-** | **-** | **+** | **-** | **-** | **+** | **-** | **-** | **-** | **-** | **-** | **-** | **-** | | **-** | | | **-** | | **-** | **+** | **+** | **+** | **+** |
| O45:H19 | **1** | **-** | **+** | **+** | **+** | **-** | **+** | **-** | **+** | **+** | **+** | **-** | **-** | **+** | **-** | **-** | **+** | **-** | **-** | **-** | **-** | **-** | **-** | **-** | | **-** | | | **-** | | **-** | **-** | **+** | **-** | **+** |
| O45:H19 | **1** | **-** | **+** | **+** | **-** | **-** | **+** | **-** | **-** | **-** | **-** | **-** | **-** | **-** | **-** | **-** | **+** | **-** | **-** | **-** | **-** | **-** | **-** | **-** | | **-** | | | **-** | | **-** | **+** | **+** | **-** | **+** |
| O45:H21 | **1** | **-** | **-** | **-** | **+** | **-** | **-** | **-** | **-** | **+** | **+** | **-** | **+** | **+** | **+** | **+** | **-** | **-** | **-** | **-** | **-** | **+** | **-** | **-** | | **-** | | | **-** | | **-** | **+** | **+** | **+** | **+** |
| O45:H21 | **1** | **-** | **+** | **+** | **+** | **-** | **+** | **-** | **+** | **+** | **+** | **-** | **+** | **+** | **-** | **+** | **+** | **-** | **-** | **-** | **-** | **+** | **-** | **-** | | **-** | | | **-** | | **-** | **+** | **+** | **+** | **+** |
| O45:H28 | **1** | **-** | **-** | **-** | **-** | **-** | **+** | **-** | **-** | **-** | **-** | **-** | **-** | **-** | **-** | **-** | **+** | **-** | **-** | **-** | **-** | **-** | **-** | **-** | | **-** | | | **-** | | **-** | **+** | **+** | **-** | **+** |
| O45:H38 | **1** | **-** | **+** | **+** | **+** | **-** | **+** | **-** | **-** | **+** | **+** | **-** | **+** | **+** | **+** | **-** | **+** | **-** | **-** | **-** | **-** | **-** | **-** | **-** | | **-** | | | **-** | | **-** | **-** | **+** | **+** | **+** |
| O45:H38 | **1** | **-** | **+** | **+** | **+** | **-** | **+** | **-** | **-** | **+** | **+** | **-** | **+** | **+** | **+** | **-** | **+** | **-** | **-** | **-** | **-** | **+** | **-** | **-** | | **-** | | | **-** | | **-** | **-** | **+** | **+** | **+** |
| O45:H38 | **1** | **-** | **+** | **+** | **+** | **-** | **+** | **-** | **-** | **+** | **+** | **-** | **+** | **-** | **+** | **+** | **+** | **-** | **-** | **-** | **-** | **+** | **-** | **-** | | **-** | | | **-** | | **-** | **+** | **+** | **+** | **+** |
| O45:H38 | **1** | **-** | **+** | **+** | **+** | **-** | **+** | **-** | **-** | **+** | **+** | **-** | **+** | **+** | **+** | **+** | **+** | **-** | **-** | **-** | **-** | **+** | **-** | **-** | | **-** | | | **-** | | **-** | **-** | **+** | **+** | **+** |
| O45:H38 | **1** | **-** | **+** | **+** | **+** | **-** | **+** | **-** | **-** | **-** | **-** | **-** | **-** | **-** | **-** | **-** | **-** | **-** | **-** | **-** | **-** | **-** | **-** | **-** | | **-** | | | **-** | | **-** | **-** | **+** | **-** | **+** |
| O45:HNT | **1** | **-** | **+** | **+** | **+** | **-** | **+** | **-** | **-** | **+** | **+** | **-** | **+** | **+** | **+** | **-** | **+** | **-** | **-** | **-** | **-** | **-** | **-** | **-** | | **-** | | | **-** | | **-** | **-** | **+** | **+** | **+** |
| O45:HNT | **2** | **-** | **+** | **+** | **+** | **-** | **+** | **-** | **-** | **-** | **-** | **-** | **-** | **-** | **-** | **-** | **-** | **-** | **-** | **-** | **-** | **-** | **-** | **-** | | **-** | | | **-** | | **-** | **-** | **+** | **-** | **+** |
| O45:HNT | **1** | **-** | **+** | **+** | **+** | **-** | **+** | **-** | **-** | **-** | **-** | **-** | **+** | **-** | **-** | **-** | **+** | **-** | **-** | **-** | **-** | **-** | **-** | **-** | | **-** | | | **-** | | **-** | **-** | **+** | **-** | **+** |
| O45:HNT | **1** | **-** | **+** | **+** | **+** | **-** | **+** | **-** | **-** | **+** | **+** | **-** | **+** | **+** | **+** | **+** | **+** | **-** | **-** | **-** | **-** | **-** | **-** | **-** | | **-** | | | **-** | | **-** | **+** | **-** | **+** | **+** |
| O45:HNT | **1** | **-** | **+** | **+** | **+** | **-** | **+** | **-** | **-** | **+** | **+** | **-** | **+** | **+** | **+** | **+** | **+** | **-** | **-** | **-** | **-** | **+** | **-** | **-** | | **+** | | | **-** | | **+** | **+** | **+** | **+** | **+** |
| O45:HNT | **1** | **-** | **+** | **+** | **-** | **-** | **+** | **-** | **-** | **+** | **+** | **-** | **+** | **-** | **-** | **+** | **+** | **-** | **-** | **-** | **-** | **+** | **-** | **-** | | **-** | | | **-** | | **-** | **-** | **+** | **+** | **+** |
| O45:HNT | **1** | **-** | **+** | **+** | **+** | **-** | **+** | **-** | **-** | **+** | **+** | **-** | **+** | **+** | **+** | **+** | **+** | **-** | **-** | **-** | **-** | **+** | **-** | **-** | | **-** | | | **-** | | **-** | **+** | **+** | **+** | **+** |
| O45:HNT | **1** | **-** | **+** | **+** | **+** | **-** | **+** | **-** | **-** | **+** | **+** | **-** | **-** | **+** | **-** | **-** | **+** | **-** | **-** | **-** | **-** | **-** | **-** | **-** | | **-** | | | **-** | | **-** | **+** | **+** | **+** | **+** |
| O45:HNT | **1** | **-** | **+** | **+** | **+** | **-** | **+** | **-** | **-** | **+** | **+** | **-** | **-** | **+** | **-** | **-** | **+** | **-** | **-** | **-** | **-** | **-** | **-** | **-** | | **-** | | | **-** | | **-** | **-** | **+** | **+** | **+** |
| O45:HNT | **1** | **-** | **+** | **+** | **+** | **-** | **+** | **-** | **-** | **+** | **-** | **-** | **-** | **+** | **-** | **-** | **-** | **-** | **-** | **-** | **-** | **-** | **-** | **-** | | **-** | | | **-** | | **-** | **-** | **+** | **+** | **+** |
| O45:HNT | **1** | **-** | **-** | **+** | **-** | **-** | **+** | **-** | **+** | **+** | **+** | **-** | **+** | **+** | **+** | **+** | **+** | **-** | **-** | **-** | **-** | **+** | **-** | **-** | | **-** | | | **-** | | **+** | **-** | **+** | **+** | **+** |
| **SEROTYPE** | **No.of Isolate** | ***eaeA*** | ***hlyA*** | ***saa*** | ***subA*** | ***katP*** | ***espP*** | ***etpD*** | ***pagC*** | ***sen (Z4326)*** | ***ent/ espL2*** | ***efa1 (Z4332)*** | ***efa1 (Z4333)*** | ***nleB*** | ***nleE*** | ***nleG2-3*** | ***nleG6-2*** | ***nleG5-2*** | ***nleG9*** | ***nleG*** | ***nleF*** | ***nleH1-2*** | ***nleA*** | ***nleG2-1*** | | ***nleB2*** | | | ***nleC*** | | ***nleD*** | ***nleH1-1*** | ***ter-C*** | ***ureC*** | ***iha*** |
|  |  |  |  |  |  |  |  |  |  |  |  |  |  |  |  |  |  |  |  |  |  |  |  |  | |  | | |  | |  |  |  |  |  |
| **TOTAL (22)** | **140** | **17** | **127** | **115** | **53** | **14** | **111** | **11** | **75** | **51** | **48** | **15** | **40** | **46** | **40** | **43** | **47** | **27** | **23** | **3** | **7** | **38** | **17** | **20** | | **22** | | | **17** | | **29** | **39** | **112** | **78** | **131** |
| **% Positive** |  | **12,1** | **90,7** | **82,1** | **37.9** | **10,0** | **79,3** | **7,9** | **53,6** | **36,4** | **34,3** | **10.7** | **28,6** | **32,9** | **28,6** | **30,7** | **33,6** | **19,3** | **16,4** | **2,1** | **5,0** | **27,1** | **12,1** | **14,3** | | **15,7** | | | **12,1** | | **20,7** | **27,9** | **80,0** | **55,7** | **93,6** |
|  |  |  |  |  |  |  |  |  |  |  |  |  |  |  |  |  |  |  |  |  |  |  |  |  | |  | | |  | |  |  |  |  |  |
|  |  |  |  |  |  |  |  |  |  |  |  |  |  |  |  |  |  |  |  |  |  |  |  |  | |  | | |  | |  |  |  |  |  |

**NB: ^a^Serotypes in bold have been identified previously as human pathogens causing diarrhoea, bloody diarrhoea and HUS**
